# Proton-coupled electron transfer dynamics in the alternative oxidase†

**DOI:** 10.1039/d4sc05060f

**Published:** 2024-10-11

**Authors:** Patricia Saura, Hyunho Kim, Adel Beghiah, Luke Young, Anthony L. Moore, Ville R. I. Kaila

**Affiliations:** a Department of Biochemistry and Biophysics, Stockholm University Stockholm 10691 Sweden ville.kaila@dbb.su.se; b Biochemistry and Biomedicine, School of Life Sciences, University of Sussex Falmer Brighton BN1 9QG UK

## Abstract

The alternative oxidase (AOX) is a membrane-bound di-iron enzyme that catalyzes O_2_-driven quinol oxidation in the respiratory chains of plants, fungi, and several pathogenic protists of biomedical and industrial interest. Yet, despite significant biochemical and structural efforts over the last decades, the catalytic principles of AOX remain poorly understood. We develop here multi-scale quantum and classical molecular simulations in combination with biochemical experiments to address the proton-coupled electron transfer (PCET) reactions responsible for catalysis in AOX from *Trypanosoma brucei*, the causative agent of sleeping sickness. We show that AOX activates and splits dioxygen *via* a water-mediated PCET reaction, resulting in a high-valent ferryl/ferric species and tyrosyl radical (Tyr220˙) that drives the oxidation of the quinol *via* electric field effects. We identify conserved carboxylates (Glu215, Asp100) within a buried cluster of ion-pairs that act as a transient proton-loading site in the quinol oxidation process, and validate their function experimentally with point mutations that result in drastic activity reduction and p*K*_a_-shifts. Our findings provide a key mechanistic understanding of the catalytic machinery of AOX, as well as a molecular basis for rational drug design against energy transduction chains of parasites. On a general level, our findings illustrate how redox-triggered conformational changes in ion-paired networks control the catalysis *via* electric field effects.

## Abstract 2

Here we study mechanistic principles of the membrane-bound alternative oxidase (AOX) from *Trypanosoma brucei*, elucidating how AOX activates dioxygen to drive quinol oxidation by proton-coupled electron transfer reactions and electric field effects.

## Graphical abstract

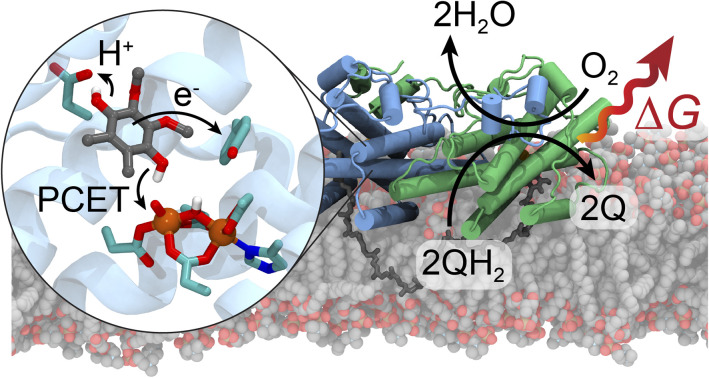

## 1 Introduction

**Fig. 1:**
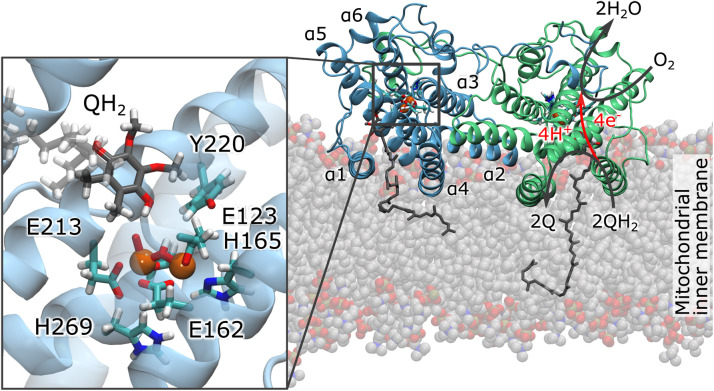
Structure and function of AOX from *Trypanosoma brucei* (PDB ID: 3VV9):^5^ the membrane-bound AOX dimer catalyzes O_2_-driven oxidation of quinol (QH_2_) to quinone (Q). (Inset) The active site comprising a di-iron core, surrounding conserved histidine and glutamate residues, and the catalytically important Tyr220. The modeled quinol bound to the active site is based on MD simulations (the isoprenoid tail is shown in transparent for clarity).

Cellular respiration is catalyzed by transmembrane (TM) redox-driven proton pumps that generate a proton motive force (pmf) across a biological membrane, driving ATP synthesis and active transport.^1,2^ Most organisms power their respiratory chains with terminal heme-copper oxidoreductases (HCOs) that reduce O_2_ to water and transduce the free energy into a pmf. However, several plants, fungi, and protists express a non-proton motive alternative oxidase (AOX) that catalyzes a quinol (QH_2_)-driven reduction of molecular oxygen to water, whilst dissipating the large driving force (∼2.9 V between *E*_m_(QH_2_/Q) = +90 mV and *E*_m_(O_2_/H_2_O) = +820 mV; Δ*G* = 4 × (0.82 − 0.09) = 2.92 V) into heat.^3^ In contrast to HCOs that share a similar core structure comprising several TM helices, the structurally unrelated AOX is anchored by two amphipathic helices to the negatively-charged side (N-side) of the membrane (Fig. 1). Each monomer of this 75 kDa homodimeric di-iron protein comprises six long α-helices (α1–α6) and four short α-helices (αS1–αS4, Fig. 1 and S1C†). The protein core is formed by a four-helix bundle (α2, α3, α5, α6) with the catalytic di-iron center, coordinated by conserved glutamates and histidine residues (Fig. 1).^4,5^ Similar coordination geometries can be observed in other di-iron proteins, such as methane monooxygenase (MMO)^6^ and ribonucleotide reductase (RNR).^3,7^ Spectroscopic and mutagenesis studies indicated that a tyrosyl radical is involved in the catalysis of RNR.^3,8^ Similarly, a homologous tyrosine residue (Tyr220) is also located next to the di-iron center of AOX that could participate in the catalysis (Fig. 1). Fourier-transformed infrared spectroscopic (FTIR) studies^9^ further suggested that a tyrosyl radical could also form in AOX, although the mechanistic principles underlying the quinol oxidation process still remain unclear.^10^

AOX has an important bioenergetic role in OXPHOS chains of many organisms, where it reduces levels of reactive oxygen species (ROS) by controlling the redox state of the quinol pool, protecting it against oxidative cell damage.^3,11^ In the pathogenic parasite of tsetse-flies, *Trypanosoma brucei*, the causative agent of African trypanosomiasis (sleeping sickness), AOX operates as the sole terminal oxidoreductase when the parasite resides in the bloodstream of its mammalian host,^12,13^ whereas it also functions as a terminal oxidoreductase in several phytopathogenic fungi, which threaten the world's food supply.^10,14^ As AOX is not present in mammalian respiratory chains, the design of inhibitors is thus of significant biomedical and therapeutic interest.^15^

To derive a molecular insight into the elusive proton-coupled electron transfer (PCET) reactions linked to the quinol oxidation process in the trypanosomal AOX, we combine here hybrid quantum/classical (QM/MM) free energy simulations, and density functional theory (DFT)-based cluster models with atomistic molecular dynamics (MD) simulations, site-directed mutagenesis experiments and activity assays. Our combined findings illustrate key catalytic principles and electric field effects that govern the PCET reactions linked to the O_2_-driven quinol oxidation in AOX, with mechanistic similarities to other enzymes.

## 2 Results

### 2.1 Activation and splitting of the O–O bond

**Fig. 2:**
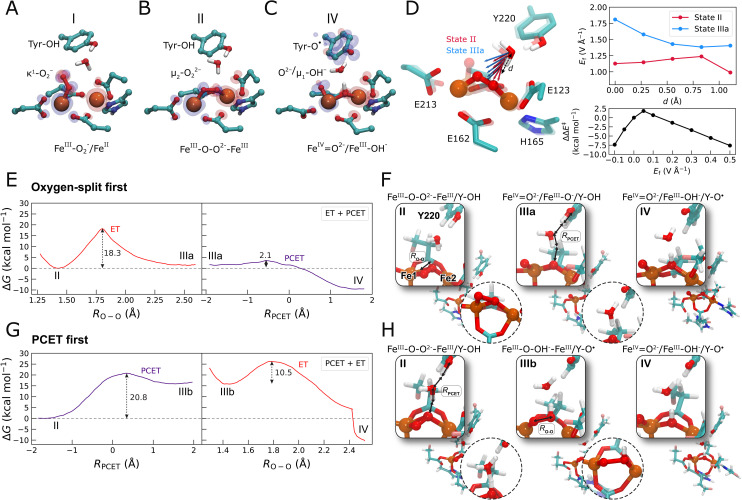
Oxygen activation and splitting reaction in the active site of AOX. (A–C) DFT models of the AOX catalytic core, showing optimized structures of (A) O_2_ bound in an (κ^1^)-mode (state I), (B) O_2_ bridging the Fe centers in a (μ_2_)-binding mode (state II), and (C) the product state of the oxygen splitting process (Fe^IV^=O^2−^/Fe^III^–OH^−^) (state IV). Only central atoms are shown. Spin density distributions are shown as transparent surfaces (α, blue; β, red). See Fig. S1A and S2† for the complete models, and Fig. S3† for QM/MM-MD simulations. (D) (Left) Intrinsic electric field (*E*_f_) vectors along the proton transfer pathway (*d*) from Tyr220 to O_2_ in state II (red arrows) and state IIIa (blue arrows) point in the direction of the proton transfer reaction. (Top right) *E*_f_ strength along the proton transfer pathway (*d*) along pathways 1 and 2, suggests that the barrier modulation in the PCET step arises from electric field effects. (Bottom right) An external *E*_f_ directed along the bridging water molecule modulates the PCET barrier along pathway 2, similarly to the intrinsic electric field (*E*_f_) along pathway 1 (see also Fig. S17†) the applied external field mimics the electric field difference (Δ*E*_f_) between states IIIa and II. (E and G) QM/MM free energy profiles of the two O_2_-splitting pathways. The reaction coordinate *R*_O–O_ represents the O–O distance, whereas *R*_PCET_ represents a linear combination of bond-breaking/forming distances from Tyr220 to oxygen *via* the bridging water molecule. Red profiles correspond to the ET step, and purple profiles to the PCET step. (E) Pathway 1: O–O bond splitting (left) followed by PCET from Tyr220 (ET + PCET). (F) Structure of the intermediates along pathway 1: II: Fe^III^–O–O^2−^–Fe^III^/Y–OH; *R*_O–O_ is shown; IIIa: Fe^IV^=O^2−^/Fe^III^–O^−^/Y–OH; *R*_PCET_ is shown; IV: Fe^IV^=O^2−^/Fe^III^–OH^−^/Y–O˙. The TS between the different steps are shown in circles. (G) Pathway 2: water-mediated PCET from Tyr220 to O_2_, followed by splitting of the O–OH^−^ bond (PCET + ET). (H) Structure of intermediates along pathway 2: II: Fe^III^–O–O^2−^–Fe^III^/Y–OH; *R*_PCET_ is shown; IIIb: Fe^III^–O–OH^−^/Fe^III^/Y–O˙; *R*_PCET_ is shown; IV: Fe^IV^=O^2−^/Fe^III^–OH^−^/Y–O˙, with TS structures shown as insets. See Fig. S2 and S5† for details of the DFT and QM/MM models.

To probe key steps along the catalytic cycle of AOX from *T. brucei*, we first modeled the initial oxygen activation and splitting reaction using QM/MM and DFT models, constructed based on snapshots from classical MD simulations or an X-ray structure of AOX, respectively (PDB ID: 3VV9,^5^Fig. 2, Table S1 and Fig. S1A†).

The DFT calculations suggest that O_2_ binds to the reduced Fe^II^/Fe^II^ core, initially in an end-on (κ^1^) binding mode coordinating one iron center (Fe_1_), followed by relaxation to the side-on (μ_2_-*trans*)-mode (Δ*G* = −0.5 kcal mol^−1^), where the dioxygen bridges both irons (Fe_1_/Fe_2_) (Fig. 2A, B and S2A†). The barrier for the end-on to side-on (κ^1^ → μ_2_) transition is moderate (Δ*G*^‡^ = 13.5 kcal mol^−1^, Fig. S2J†), and kinetically much faster than the overall experimental turnover rate (∼0.7 s^−1^/Δ*G*^‡^ = 18 kcal mol^−1^) of AOX (based on transition state theory) (see Fig. S4† for different binding modes). These binding modes are further supported by our DFT-based QM/MM molecular dynamics simulations, where the end-on (κ^1^) binding mode (Fig. S3A–C†) relaxes either into the side-on-(μ_2_)-*cis* or side-on-(μ_2_)-*trans* configuration, where a single oxygen bridges the iron core (Fig. S3D–G and S4B–D†). Recent structural studies of the inhibitor-bound AOX also support the side-on binding mode,^15^ whereas Yamasaki *et al.*^16^ found, based on QM/MM calculations, that O_2_ can also bind in an end-on mode to the iron when OH^−^ is bridged between the metals.

Both the side-on O_2_-binding modes captured in our DFT and QM/MM models show a superoxide (O_2_˙^/−^) character achieved by partial electron transfer from the Fe^2+^ centers, leading to an overall mixed valence Fe^2.5+/^Fe^2.5+^ state (Fig. 2B, S2D–G and S3A–D†). The overall binding affinity of O_2_ is around −3.0 kcal mol^−1^ (Fig. S2J†), which compares well with the experimental apparent *K*_m_ for O_2_ of around 14 μM,^10^*cf.* also ref. 17. Our QM/MM models show that the transition of side-on bound O_2_ from μ_2_-*cis* to μ_2_-*trans* increases the oxygen–oxygen distance from *ca.* 1.35 Å to 1.43 Å (Fig. S3B†), suggesting the transition from a superoxide (O_2_˙^/−^) to a peroxide-like (O_2_^2−^) species. This process is accompanied by an increase in negative charge on the oxygens (Fig. S3E and F†).

In the side-on binding mode, the dioxygen molecule forms a water-mediated hydrogen-bonded contact with the nearby conserved tyrosine residue (Tyr220) that could mediate a PCET reaction and result in the splitting of the dioxygen bond. To study the mechanisms of the O_2_ splitting reaction, we performed QM/MM free energy calculations based on energetically preferred side-on binding mode (Fig. 2 and S5,† see Methods), and by considering (1) an initial O–O bond splitting followed by PCET reaction from Tyr220, or (2) a PCET reaction followed by the O–O bond splitting. For pathway 1, where the O–O bond splitting precedes the PCET reaction, we obtain a free energy barrier of 18.3 kcal mol^−1^ (Fig. 2E) with a peroxide-like transition state (*d*_OO_ = ∼1.76 Å, Fig. 2F), which relaxes in an exergonic reaction (Δ*G* = −9 kcal mol^−1^) into a ferryl (Fe^IV^=O)/ferric hydroxo (Fe^III^–OH^−^)-species and a neutral tyrosyl radical. The water-mediated PCET reaction from Tyr220 to the peroxy-intermediate along this reaction pathway has a small reaction barrier of <5 kcal mol^−1^ (Fig. 2E). In stark contrast, when the PCET reaction precedes *via* the O–O bond splitting process (pathway 2), we obtain a significantly higher free energy barrier of 20.8 kcal mol^−1^ that results in an unstable peroxy intermediate (Fe^III^–OOH^−^, Δ*G* = 16 kcal mol^−1^) and a neutral tyrosyl radical. This peroxy-state further splits into the Fe^III^–O^−^/Fe^III^–OH^−^ state (with Δ*G*^‡^ = 10 kcal mol^−1^, Fig. 2G and H), followed by the exergonic relaxation of the Fe^III^–O^−^/Fe^III^–OH^−^ into the Fe^IV^=O^−^/Fe^III^–OH^−^ state (Fig. S5G†), leading to the same product state as in reaction pathway 1 (*cf.* also ref. 16 and refs therein). Taken together, our QM/MM calculations strongly suggest that the O–O splitting precedes the PCET reaction in AOX.

To understand the basis for the different energetics along the two proposed pathways, we investigated the electric field effects during oxygen activation using our DFT models. Interestingly, we find an intrinsic orientated electric field between Tyr220 and the Fe-bound oxygen ligand prior to the PCET step (Fig. 2D) that is significantly stronger along pathway 1 relative to pathway 2 (state IIIa *vs.* state II), suggesting that the large barrier modulation along the water-mediated PCET pathway could arise from electric field effects (Fig. 2D). This is further supported by a similar barrier reduction observed upon the application of an external electric field along the O–H bond in pathway 2 (state II → IIIb step) (Fig. 2D, bottom right) mimicking the effects of the intrinsic electric fields arising along pathway 1 (Fig. 2D, top right), suggesting that the electric fields near the active site of AOX are catalytically important (Fig. 2D and S17†). The electric field perturbation leads to a linear perturbation in the PCET barrier with the driving force, and a Brønsted slope of one (Fig. S17†).

Analysis of the spin and charge density distributions further support that the proton and electron transfer reactions from Tyr220 are coupled to the electron transfer to the O–O species, whilst the oxygen splitting leads to the oxidation of Fe_1_ (Fig. S2F, G and S5C, F†).

To experimentally assess the O_2_ reduction process, we next measured the oxygen consumption of AOX using an oxygen electrode (see Methods), where we initiated the reaction by adding 500 μM short-tailed quinol (Q_1_H_2_). We obtain a quinol-driven O_2_ consumption rate for the WT-AOX of 0.7 ± 0.04 s^−1^, which compares well with earlier estimates,^10^ and indicates that the rate-limiting barrier along the catalytic cycle is around 18 kcal mol^−1^ (based on transition state theory). These estimates fit well to the predicted free energy barriers of around 18 kcal mol^−1^ for the initial O–O spitting reaction (Fig. 2E).

### 2.2 Substrate binding and protein-membrane interactions

To probe the dynamics of AOX in the resulting ferryl state on longer timescales, we next derived atomistic force field parameters for the active site based on the DFT calculations (Table S3†). This allowed us to explore the dynamics of AOX on microsecond timescales using atomistic MD simulations (Fig. S1C and Table S2†). To this end, we embedded AOX in a POPC lipid membrane/water/ion environment and docked a quinol-molecule (Q_10_) into the active site based on a resolved structure of an inhibitor-bound AOX (PDB ID: 3W54 (ref. 5)), and modeled the isoprenoid tail in the non-polar cavity that extends to the lipid membrane (see Methods, Fig. S1C†).

During the classical MD simulations, AOX establishes several charged interactions with the phospholipid headgroups and the amphipathic helices α2/α4 (α2*/α4*) (Fig. S7†). Our MD simulations suggest that the major groove around α2/α4 (α2*/α4*) forms a larger interaction area with the lipids, although helices α1 and α4 have previously been implicated to form lipid binding sites.^5^ We observe additional charged interactions with the membrane surface, particularly around Arg93 and Arg96 (Fig. S7A and S8A–C†) that form a cluster of charged residues near the active site and could have an important functional role in quinol oxidation catalysis (see below).

**Fig. 3:**
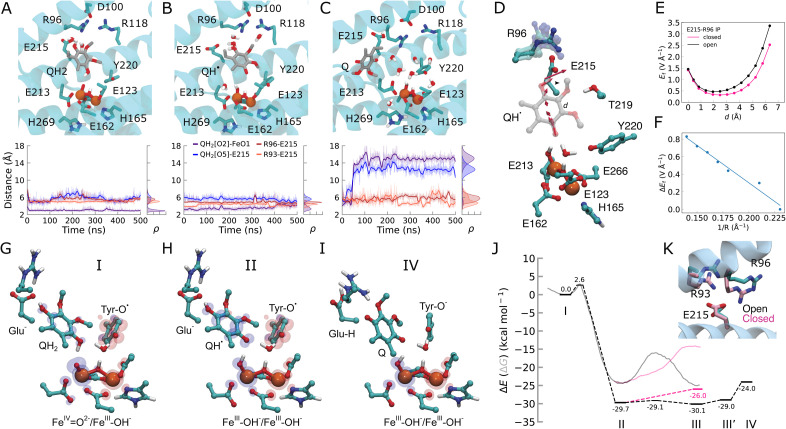
Dynamics of the quinol during the oxidation process. (A) MD snapshots showing key residues interacting with the substrate in the (A) quinol (QH_2_), (B) semiquinone (QH˙), and (C) quinone (Q) states. Distances between the Q species and key residues during the MD simulations are shown below. (D) The electric field vectors in the active site along the QH˙ species. The Glu215/Arg96 ion-pair samples closed or open conformations during MD simulations. (E) Electric field strength (in V Å^−1^) along the proton transfer pathway (*d*, quinol: O5 → O2 direction) in open (black) and closed (pink) conformations of the Glu215/Arg96 ion-pair. (F) The effect of E215/R96 ion-pair distance (*R*) on the electric field indicates a linear correlation. (G–I) DFT optimized structures and spin distributions (α, blue; β, red) of intermediate states. (J) Energetics of the quinol oxidation reaction. Dashed lines: DFT electronic energies (Δ*E*)/transparent lines: QM/MM free energies (Δ*G*, see Fig. S11L–N†) for the quinol oxidation process: PCET from QH_2_ to Fe^IV^=O^2−^ (state I) results in QH˙/Fe^III^–OH^−^ (state II). QH˙ transfers its proton to Glu215, followed by electron transfer from Q˙ to the tyrosyl radical, forming a tyrosinate anion and dissociation of the oxidized quinone (state IV). See Fig. S10† for all DFT optimized structures and for spin and charge population analysis. The black profiles correspond to an open Glu215/Arg96 ion-pair, with the closed Glu215/Arg96 ion-pair effect shown in the pink profiles (see Fig. S11† and Methods). (K) MD snapshots showing the open and closed conformation of the Glu215/Arg96/93 ion-pairs.

The quinol remains bound within the active site pocket during the MD simulations by hydrogen-bonded contacts with the ferryl oxygen and Glu215, which in turn, forms ion-pairs with Arg96 and Arg93 (Fig. 3A–C). The quinol also interacts with other polar residues around the active site (Thr219, Arg118), with a binding mode that resembles the experimentally resolved inhibitor-bound AOX structures (see Fig. S9†).^5,15^ The quinol tail protrudes into the membrane *via* a *ca.* 11 Å long cavity that starts with several non-polar residues, followed by the charged cluster at the top of the binding site (Arg93, Arg96, Asp100, Arg118, Glu215, Thr219; Fig. S8 and S12†). Cavity analysis suggests that Leu212 and Leu122 form a bottleneck within this tunnel (see also ref. 4, Fig. S8F and G†). In the apo state, the α2-helix makes a subtle bend into the quinol cavity, which decreases the distance between the leucine residues and affects the overall volume of the cavity (Fig. S8F and G†).

### 2.3 Quinol-driven oxidation dynamics

To probe the energetics linked to the quinol oxidation process, we next created QM/MM and DFT models of AOX that allowed us to probe both local and non-local effects of the rather extended substrate binding pocket (Fig. S1B, D, S10 and S11†). We obtain a rather low free energy barrier (Δ*G*^‡^ = 2.6 kcal mol^−1^ in DFT models; 3.7 kcal mol^−1^ in QM/MM) for the PCET reaction from the quinol to the active site ferryl. The reaction leads to an exergonic formation of the neutral semiquinone QH˙/ferric hydroxide (Fe^III^–OH^−^) species (*ca.* −30 kcal mol^−1^ in DFT models, −25 kcal mol^−1^ in QM/MM, Fig. 3G–J, S10, S11 and Table S1†). We next probed the dynamics of the quinol oxidation intermediates using classical MD simulations. Interestingly, the semiquinone forms a hydrogen-bonded contact with Glu215 (Fig. 3B and S12†), suggesting that Glu215 could serve as a potential proton acceptor of the distal QH˙ proton.

Our DFT calculations further reveal that the proton transfer from QH˙ to Glu215 is indeed energetically favored (Δ*E* = −0.4 kcal mol^−1^) with a small energy barrier (Δ*E*^‡^ = 0.6 kcal mol^−1^). The reaction leads to a concerted transfer of the semiquinone electron to Tyr220–O˙, forming an anionic tyrosinate (TyrO^−^) and a neutral quinone (Fig. 3G–J, S10 and S11†). This PCET process results in reorganization of the active site and partial dissociation of the Q towards the membrane (Fig. 3C, I and S12†).

Glu215 forms ion-pairs with the fully conserved Arg96 and the partially conserved Arg93 (Fig. S13†) that sample both open and closed conformations during our MD simulations (Fig. 3A–C and S12†). Moreover, we observe that the QH˙ species can enhance the opening of the Glu215–Arg96 and Glu215–Arg93 ion-pairs, which strongly modulates the energetics of the proton transfer from the QH˙ to Glu215 (Fig. 3J, K and S11†). In the open state model, we obtain a weakly exergonic proton transfer reaction from DFT calculations (−0.5 kcal mol^−1^), whereas the proton transfer is highly endergonic (8 kcal mol^−1^) in the closed conformation (Fig. 3J and K). These findings are further supported by our DFT-based QM/MM molecular dynamics simulations, in which the proton is transferred back from Glu215 to the semiquinone in the closed ion-pair conformation, whereas the proton remains bound on Glu215 in the open ion-pair state (Fig. S11I†). Finally, QM/MM free energy calculations also support that the ion-pair conformation modulates the energetics of these proton transfer reactions (Fig. 3J, S11M and N†).

To analyze the possible origin of these tuning effects, we computed electric fields at the DFT level. We find that the formation of QH˙ induces an electric displacement vector, leading towards the Glu215–Arg96/Arg93 ion-pairs that could favor its dissociation (Fig. 3D, S10D and E†). The ion-pair dissociation further increases the electric field along this pathway (Fig. 3D–F) and lowers the barrier for the proton transfer reaction (Fig. 3J and S11G†). We note that during the earlier steps of the catalytic cycle, electric field vectors also point along the Tyr220–O_2_ axis (Fig. 2D), similar to the electric fields between the QH_2_ and the ferryl species that form during the quinol oxidation process (Fig. S10D and E†), suggesting that electric field effects could modulate these reaction steps. Taken together, these findings suggest that AOX may employ internal electric field effects that control the barrier and driving forces for the redox-state dependent PCET reactions. Rather than from electrostatic pre-organization,^18^ this effect could arise from dynamic changes at the active site, where the quinone chemistry and reduction of the di-iron center give rise to electric field effects. Interestingly, similar ion-pair controlled proton transfer reactions have recently been described in several other enzymes,^19^ including cytochrome *c* oxidase,^20^ complex I,^21^ photosystem II,^22^ and Hsp90 ^23^ (see Discussion). Electric field effects have also been proposed to control catalysis in other systems (*cf.* ref. 24 and refs therein).

We next probed the dynamics of the resulting oxidized quinone and protonated Glu215 in our classical MD simulations. Our results show that protonation of Glu215 induces dissociation of the Glu215–Arg96 ion-pair on the microsecond timescale, an effect that propagates to the adjacent ion-pairs (Glu215/Arg93 and Arg118/Asp100, Fig. 3 and S12†). These redox- and protonation-driven conformational changes lead to an influx of water molecules into the active site (Fig. S14A and B†) that could contribute to dissociation of the quinone towards the membrane, as observed in our MD simulations, as well as the re-protonation of Tyr220 (see below), which is required for the next turnover cycle. We note that the hydration level of the quinone cavity is also sensitive to the charge state of the surrounding groups, as well as to the specific ligand state (Fig. S14A and B†).

**Fig. 4:**
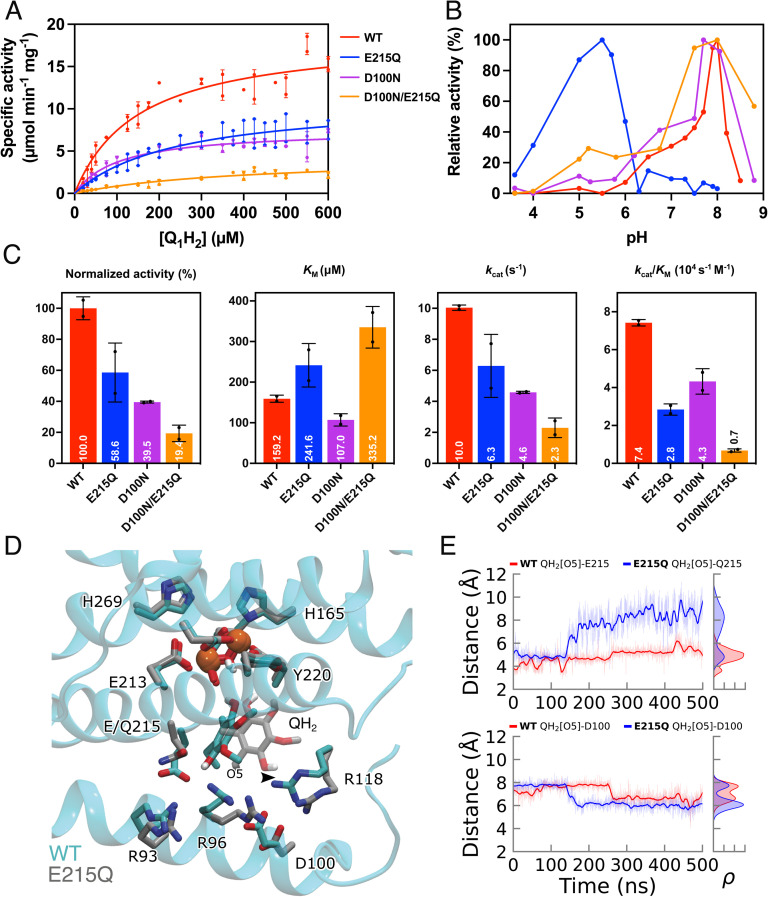
Functional assays of AOX. (A) Michaelis–Menten plot of the AOX activity of the WT (red), E215Q (blue), D100N (purple), and D100N/E215Q (orange) constructs. (B) AOX activity measured at different pHs using 150 μM QH_2_. The relative activity is normalized relative to each construct, with a peak value set to 100%. (C) Enzymatic activity and Michaelis–Menten parameters of AOX determined for purified WT and mutants at pH = 7.5. (D) MD snapshot showing the QH_2_ binding mode in the WT (cyan) and the E215Q mutant (grey). (E) Distance between QH_2_ O5 and residues Glu/Gln215 (top) and Asp100 (bottom) from MD simulations.

To experimentally validate the functional role of this putative protonation site, we created the E215Q variant by site-directed mutagenesis experiments, a substitution that mimics the protonated form of Glu215, as suggested by our MD simulations (Fig. 3 and S12†). Interestingly, the E215Q variant shows around 60% of the WT activity (Fig. 4A and C), and the binding affinity for the quinol (Q_1_H_2_) is reduced (*K*_m_ = 159 μM for WT, *K*_m_ = 242 μM for E215Q), supporting that the residue is functionally central for the quinol oxidation process. We observe that the maximum activity for WT-AOX at pH = 8 is strongly shifted in the E215Q variant to pH = 5.5 that could reflect the apparent p*K*_a_ of the putative protonation site (see below).

Our MD simulations of the mutant further suggest that the E215Q substitution shifts the quinol binding mode towards Asp100, which could function as an alternative proton-acceptor in the E215Q variant (Fig. 4D, E and S15†). Moreover, the substitution leads to an increase in the Gln215–Arg96 (and Gln215–Arg93) distance, as well as an increase in water molecules near the quinol (Fig. S14 and S15†). The DFT calculations suggest that the proton transfer from QH˙ to Asp100 is also feasible (Δ*E*^‡^ = 15 kcal mol^−1^, Δ*E* = 10 kcal mol^−1^, see Fig. S16†), although the barrier and thermodynamic driving force are somewhat higher as compared to the proton transfer to Glu215. A possible reason is that Asp100 is shielded by both Arg96 and Arg118, for which we do not observe a similar redox-dependent conformational switching as for the Glu215/Arg93 ion-pair.

To experimentally probe the alternative proton acceptor sites, we prepared the D100N and E215Q/D100N variants and measured their coupled O_2_:quinol oxidoreductase activity. The D100N variant has a lowered activity of 40% as compared to the WT, with a 0.2-pH unit shift of the activity (Fig. 4A–C), whereas the E215Q/D100N double mutant shows a significant inhibitory effect (19% of the WT activity), consistent with the primary role of Glu215 as the primary proton acceptor (Fig. 4), but also supporting that Asp100 is central for tuning the p*K*_a_ of this transient protonation site.

### 2.4 Completing the catalytic cycle

PCET to the active site and to the putative proton-loading site results in dissociation of the oxidized quinone towards the membrane by a subtle conformational change around helices α2/α4 (Fig. 3C and S12†). Our MD simulations of the emerging apo state show a substrate cavity filled with water molecules (Fig. S14A and B†) that could allow for a water-mediated proton transfer between Glu215 and Tyr220. To probe the final steps along the reaction cycle, we placed a new quinol molecule in the substrate pocket, and probed its behavior with MD simulation and DFT calculations (Fig. S12 and S14C–E†). Binding of the new quinol substrate induces a partial drying of the Q-cavity, whereas the quinol establishes contacts with both the Fe^III^–OH^−^/Fe^III^–OH^−^ and Glu215 based on our MD simulations (Fig. S12†). Our DFT calculations suggest that quinol oxidation by PCET to Fe^III^–OH^−^ results in a Fe^II^–H_2_O–Fe^III^–OH^−^ species, whilst proton transfer from QH˙ to Glu215 forms an anionic semiquinone (Q˙^/−^) and a protonated carboxylic acid (Fig. S14C†), similar as in the first quinol oxidation step. We note that the resulting water ligand in the Fe^II^–H_2_O–Fe^III^–OH^−^ state binds rather weakly to the iron, leading to the Fe^II^/Fe^III^–OH^−^ form upon dissociation. The DFT calculations also suggest that electron transfer from Q˙^/−^ to the ferric iron results in an Fe^II^/Fe^II^–H_2_O state, whereas dissociation of both Q and the H_2_O ligand could initiate the next catalytic cycle (Fig. S14C and D†). The second quinol molecule is thus required to re-reduce the di-ferric (Fe^III^/Fe^III^) state into the fully reduced di-ferrous (Fe^II^/Fe^II^) form(*cf.* ref. 3, 25 and 26).

## 3 Discussion

**Fig. 5:**
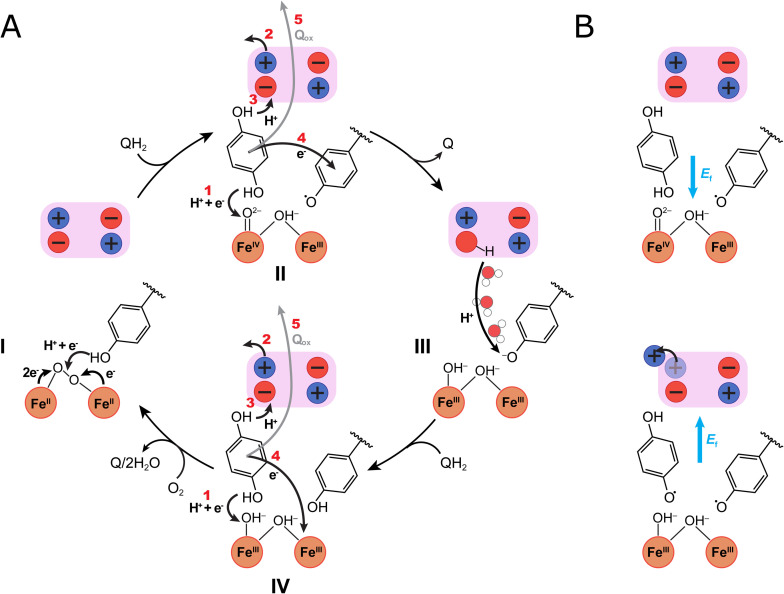
Putative catalytic cycle of AOX. The figure shows the di-iron core of the active site (orange spheres), Tyr220, and the ion-pair cluster of the transient protonation site (pink box), with redox and ligand states suggested by DFT calculations. (A) The catalysis starts from (I) the Fe^II^/Fe^II^/Tyr–OH state that binds molecular oxygen. Oxygen splitting and PCET from Tyr220 results in (II) a ferryl state and tyrosyl radical (Fe^IV^=O^2−^/Fe^III^–OH^−^/Tyr–O˙) that drive the quinol oxidation process: (1) PCET from QH_2_ to the Fe^IV^=O^2−^ produces a semiquinone (QH˙) and a di-ferric state (Fe^III^–OH^−^/Fe^III^–OH^−^/Tyr–O˙); (2) ion-pair dissociation, induces (3) an electric field (see panel B) that points towards the protonation site, which mediates the proton transfer from QH˙ to Glu215; (4) reduction of the Tyr–O˙ to a tyrosinate (Tyr–O^−^); and (5) quinone (Q) dissociation from the binding pocket. (III) Water molecules mediate re-protonation of Tyr–O^−^ from the protonation site, followed by (IV) binding of a new quinol (QH_2_): (1) PCET from the QH_2_ leads to reduction of the active site (Fe^II^–OH_2_/Fe^III^–OH^−^/Tyr–O^−^), followed by (2) ion-pair opening; (3) proton transfer to Glu215; (4) electron transfer from the QH˙ to the active site, and (5) dissociation of the Q, formation of the fully reduced Fe^II^/Fe^II^ state, and initiation of a new catalytic cycle. (B) Electric fields (*E*_f_, blue arrows) during the quinol oxidation process. (Top) In the QH_2_ state, an electric field points towards the di-iron site that drives PCET from QH_2_ to the iron. (Bottom) After QH˙ forms, opening of the Glu215/Arg96–93 ion-pair creates an electric field in the direction of the proton-loading site, that drives pT from QH˙ to Glu215. See Fig. 3D, E and S10D† for further details of the field calculations.

The catalytic cycle of AOX is initiated by the activation and splitting of molecular oxygen, resulting in a ferryl state with a high oxidation potential, which in turn drives the stepwise oxidation of quinol substrates to regenerate the resting reduced form. Our combined findings suggest that oxygen binds to the di-ferrous state (Fe^II^/Fe^II^) resulting in the O–O bond splitting, followed by a tyrosine-mediated PCET reaction that creates a neutral tyrosyl radical (TyrO˙) and a high valent ferryl/ferric (Fe^IV^=O^2−^/Fe^III^–OH^−^) intermediate (Fig. 5). Similar radical intermediates are also found in several other oxygen activating enzymes such as cytochrome *c* oxidase,^17^ RNR,^8^ and photosystem II.^22^

The involvement of the high-valent iron species in the catalytic cycle of AOX has remained unclear,^3^ although we note that FTIR studies showed the presence of an organic radical at 1554 cm^−1^ in an oxidized state, that could be attributed to a tyrosine residue.^9^ This vibrational band disappears upon reduction of the active site, supporting that it is redox active, and consistent with our DFT models. Tyrosyl radicals have been described in other di-iron proteins such as RNR,^7,27–29^ and recent studies suggest that hydrogen-bonded and possibly ion-paired networks could also facilitate PCET reaction in RNR.^29^

The quinol oxidation in AOX is driven by the high-valent ferryl and TyrO˙ species, which catalyze the stepwise PCET reaction to the iron center, and proton transfer to Glu215 on the other side of the quinol binding pocket. Protonation of the Glu215 is modulated by the opening of the Glu215/Arg96/93 ion-pairs in a reaction that could be sensitive to the electric field created by the semiquinone species itself (Fig. S10†). Our calculations also indicate that electric fields could drive the PCET reactions to the active site (Fig. 3D–F). We note that the arrangement of the Tyr220/di-iron core, as well as the Glu215–Arg96/93 ion-pair, are conserved across the different AOX isoforms, suggesting that similar electric field effects could be catalytically relevant across the AOX family. Similar ion-pair driven protonation reactions are found in several other enzymes, including cytochrome *c* oxidase,^20^ complex I,^21^ and photosystem II,^22^ suggesting that electrostatic effects may form general gating motifs in enzymes.^19,30^

Protonation of Glu215 was found to induce conformational changes in a network of charged residues, favoring the release of the oxidized quinone to the membrane. Interestingly, prior FTIR studies^9^ support the redox-triggered protonation of a carboxylate during the catalysis, which is consistent with the suggested role of Glu215 and its surrounding network of charged residues as a redox-triggered protonation site, as well as previous mutagenesis studies also supporting the functional role of this region.^10^ This proposed site has an interesting functional similarity to the proton-loading site (PLS) of cytochrome *c* oxidase,^20,31–35^ which stores the protons for pumping across that membrane, and may also undergo a redox-driven conformational change.^20^

Substitutions of Glu215 and/or Asp100 to their respective amines in AOX increase the total charge of the putative protonation site, which in turn, is expected to decrease its proton affinity. We suggest that the drastically shifted pH-optimum of E215Q reflects the intrinsic apparent p*K*_a_ of the protonation site, which is particularly sensitive to the substitution of Glu215 that could act as a proton acceptor during the quinol oxidation. This putative protonation site could also re-protonate the anionic Tyr220, when its p*K*_a_ is higher than that of external pH, but lower than that of Tyr220 (∼10), as expected for the WT AOX, based on the pH dependence of the activity (Fig. 4). In contrast, for the E215Q variant, the p*K*_a_ of this cluster is <7, suggesting that deprotonation of QH˙ takes place to the bulk solvent, with re-protonation of the active site mediated, *e.g.*, upon the quinone release. Our MD simulations suggested the E215Q substitutions also perturb the ion-pair dynamics, and affect the energetics of the proton release step both to the bulk and to Tyr220. The proposed protonation site could thus catalyze the deprotonation of the quinol that otherwise becomes rate-limiting for the overall reaction.

## 4 Conclusions

We have studied here the catalytic principles and functional dynamics of the PCET reactions linked to the quinol oxidation in the AOX, using multi-scale simulations in combination with mutagenesis studies and functional assays. Our findings suggest that oxygen is activated and split by the di-iron core, producing a highly oxidizing ferryl/ferric state and tyrosyl radical (Tyr220) that drive the stepwise oxidation of the quinol by electric field effects. In addition to the direct PCET between the quinol and the active site, we identified a conserved carboxylate cluster, comprising Glu215 and Asp100 that are in ion-paired contacts with conserved arginine residues on the distal side of substrate binding pocket. These carboxylates are suggested to form a transient proton acceptor site, regulating catalysis as well as proton and substrate release. The p*K*_a_ of this protonation site is modulated by redox-driven conformational changes in ion-pairs that participate in deprotonation of the quinol by changes in electric fields. Similar redox-driven protonation dynamics linked to the conformational changes in ion-pairs have recently been described in several enzymes,^19,20,22^ suggesting that they could comprise general functional motifs in redox catalysis. Our combined findings highlight key steps in the O_2_-driven quinol oxidation process, providing functional insight into AOX. We identify key residues in the substrate cavity that are essential for substrate binding and catalysis, establishing a basis for rational design of new phytopathogenic and anti-parasitic inhibitors, and molecular insight for the development of engineered AOX for the treatment of human mitochondrial dysfunctions.^36^

## Data availability

The data supporting this article, including materials and methods, figures showing details of DFT and MD models, derived force field parameters, data for activity assays of WT and engineered AOX can be found in the ESI.†

## Author contributions

V. R. I. K. conceptualized the study; P. S., H. K., and A. B. curated the data; P. S., H. K., A. B., L. Y., A. L. M. and V. R. I. K. analysed data; P. S., H. K., A. B., V. R. I. K. developed methods; P. S., H. K., A. B. performed investigations and prepared visualisations; V. R. I. K. supervised the project; V. R. I. K. and A. L. M. acquired funding; V. R. I. K., P. S., H. K., and A. B. wrote the manuscript, with contributions from all authors. All authors have reviewed and edited the manuscript, and given approval to the final version.

## Conflicts of interest

A. L. M. holds patents and financial interests in the development of phytopathogenic fungicides. The other authors declare no competing financial interest.

## Supplementary Material

SC-OLF-D4SC05060F-s001
