# Supplementary material for: Proton-coupled electron transfer dynamics in the alternative oxidase
Source: Chem Sci. 2024 Oct 11;15(44):18572–80. doi: 10.1039/d4sc05060f (PMC11492382; doi:10.1039/d4sc05060f)
Supplement: SC-015-D4SC05060F-s001 [file SC-015-D4SC05060F-s001.pdf]

Supplementary Information (SI) for Chemical Science.
This journal is © The Royal Society of Chemistry 2024

Supporting Information:
Proton-Coupled Electron Transfer Dynamics in the Alternative Oxidase

Patricia Saura[1,‡], Hyunho Kim[1,‡], Adel Beghiah[1,‡], Luke Young[2], Anthony L. Moore[2], Ville R. I. Kaila[1,*]

Content:

Material and Methods

Figure S1. DFT, MD, and QM/MM models.
Figure S2. Optimized DFT models for the oxygen splitting reaction.
Figure S3. Dynamics of the oxygen binding modes at the QM/MM level.
Figure S4. Exploration of different O_2-binding modes at the di-iron site of AOX from DFT models.
Figure S5. Energetics of the oxygen activation process at the QM/MM level.
Figure S6. QM models used for the benchmarking calculations.
Figure S7. AOX-membrane interactions.
Figure S8. Quinol binding cavity.
Figure S9. Structural comparison of inhibitor-bound crystal structures and MD simulations.
Figure S10. Optimized DFT models for the first quinol oxidation reaction.
Figure S11. Energetics of the quinol oxidation process at the QM/MM level.
Figure S12. Analysis of distances from MD simulations in WT-AOX.
Figure S13. Multiple sequence alignment.
Figure S14. Hydration dynamics and final steps of the reaction cycle.
Figure S15. Analysis of distances from MD simulations in the E215Q-AOX variant.
Figure S16. Energetics of proton transfer in the E215Q mutant.
Figure S17. Electric field effects on the oxygen activation catalysis.
Figure S18. Purification and characterization of WT and mutant constructs of AOX.

Table S1. List of DFT models and modeled oxidation and ligand states.
Table S2. List of atomistic MD simulations.
Table S3. Derived force field parameters for the active site of AOX.
Table S4. Primers for the E215Q and D100N variants.
Table S5. Benchmarking the DFT calculations for a di-radical model.
Table S6. Benchmarking the DFT calculations for the quinol oxidation reaction.
Table S7. Benchmarking the DFT calculations for the oxygen splitting reaction.

SI References

Materials and Methods

Classical molecular dynamics simulations

Atomistic MD simulations of the dimeric form of AOX were performed in different oxidation/protonation states of the catalytic cycle (Table S2). The protein was modeled based on the crystal structure of alternative oxidase from *T. brucei* (PDB ID:3VV9)[1] that was embedded in a POPC lipid membrane and solvated with TIP3P water molecules, and neutralized with 150 mM NaCl. The quinol substrate was modeled based on the coordinates of inhibitor-bound crystal structures (PDB IDs: 3VVA and 3W54),[1] by aligning the quinol headgroup to the inhibitor position, and modeling the tail to fit in the protein cavity, identified using cavity analysis with CAVER 3.0.[2] The final MD model comprised *ca.* 140,000 atoms. MD simulations were performed using NAMD v. 2.13.[3]

MD simulations were performed in duplicates using an *NPT* ensemble with constant pressure (1 bar) and temperature (310 K), using a 2 fs timestep, and treating the long-range electrostatic interactions with the Particle Mesh Ewald (PME) method with a grid separation of 1 Å. The CHARMM36m[4] force field was used to simulate lipids, water molecules, and the protein, whereas force field parameters for protein cofactors, quinol, semiquinone, and quinone, were derived based on DFT calculations. To this end, a DFT model comprising the di-iron center, E123, E162, E213, E266, and H165, and the oxygen/hydroxy/water ligand was optimized at the B3LYP-D3 level (Table S1, Fig. S1).[5] Force constants were derived from the molecular Hessian, estimated at the B3LYP-D3/def2-SVP/ε=4 level,[5–7] and atomic partial charges were calculated by the restrained electrostatic potential (RESP) at the B3LYP-D3/def2-TZVP/ε=4 level.[5,6,8] A two-step RESP charge optimization was employed with the charges of the sidechain (C_β and the connected hydrogens) constrained to their CHARMM36m force field values.[4] The active site was parametrized in the $Fe^{IV}=O^{2-}/Fe^{III}-OH^-$, Fe^{III}/Fe^{III}, Fe^{II}/Fe^{III}, and Fe^{II}/Fe^{II} states. Force field parameters for the co-factors are reported in Table S3.

DFT calculations

DFT models of the active site of AOX were constructed based on the crystal structure of AOX from *T. brucei* (PDB ID:3VV9).[1] The active site models comprised the di-iron core, and protein residues of the first coordination sphere (E123, E162, E213, E266 H165), second sphere protein residues (N161, Y220, W247, I262, D265, H269), as well as a bridging water molecule, and dioxygen. H269 was modeled with both ε- and δ-nitrogen protonated. The residues were cut at the C_α-C_β bond and the C_β atoms were saturated with hydrogen atoms. The DFT models comprised 136-137 atoms (Table S1, Fig. S1, S2). DFT models were also constructed to study the quinol oxidation reactions based on snapshots extracted from the classical MD simulations (Table S2). These DFT models contained the di-iron core and its first coordination sphere ligands, protein residues (Y220, N161, D265, H269, E215, T219, L122, L212), as well as the quinol headgroup, comprising in total 195-205 atoms (Table S1, Fig. S1, S10, S14C). The effect of the closed E215/R93 ion-pair was probed by including R93 in the optimized structures. The models were minimized with the C_β positions fixed, while keeping the rest of the residues fixed to their respective positions obtained from a smaller subsystem (with E215/T219/R96/Q and water molecules). R93 was then added to the full system, followed by single point energy calculations. To probe the energetics of the proton transfer in the E215Q variant, DFT models were built based on the classically relaxed snapshots of the E215Q system. These models included the Q headgroup, Y220, Q215, D100, R118, R96, L112, A216, T219, and 7 water molecules, leading to a DFT model with 148 atoms (Fig. S16).

All DFT geometry optimizations were performed at the B3LYP-D3/def2-SVP(C,H,N,O)/def2-TZVP(Fe) level, and single point energies were calculated at the B3LYP-D3/def2-TZVP/ε=4.[5–8] The C_β positions were fixed during the structure optimizations, and solvation effects were modeled using the COSMO model[9,10] with ε=4. The spin-flip broken symmetry approach was employed to treat the spin energetics of the di-iron center, by anti-ferromagnetically coupling the two irons.[11,12] Reaction energetics were obtained by reaction pathway optimization, followed by optimization of transition states. Thermodynamic corrections were estimated based on the molecular Hessian computed at the B3LYP-D3/def2-SVP(C,H,N,O)/def2-TZVP(Fe)/ε=4 level. Electric field effects on the quinol substrate were evaluated at the DFT level by performing single-point energy calculations at the B3LYP-D3/def2-TZVP level.

The DFT calculations were benchmarked using different density functionals with varying amount of exact exchange or approximations to account for long-range charge transfer effects as well as at the correlated random-phase approximation (RPA)/def2-TZVPP level.[13] Benchmarking calculations were performed for models containing i) the $QH^\bullet$/Y220-$O^\bullet$ di-radical system with protein residues and water molecules (*N*=148

atoms), ii) for the QH_2 oxidation process (N=160 atoms), and iii) for the oxygen splitting reaction (N=137 atoms). The benchmarking calculations are shown in Tables S5-S7 and Fig. S6. The DFT and RPA calculations were performed using TURBOMOLE versions 7.2-7.5.[14]. Coordinates of the optimized DFT models are available in the Zenodo repository (10.5281/zenodo.13767891).

QM/MM calculations

Hybrid quantum/classical (QM/MM) models were built based on the classically relaxed MD snapshots of the full AOX dimer and its surroundings. The QM/MM models were trimmed to a *ca.* 40,000 atom system, including the protein and its surroundings (Fig. S1D). To study the oxygen binding and activation process, the selected QM region comprised the dioxygen molecule, the di-iron core, protein residues around the catalytic site (E123, E162, E213, E266, H165, N161, D265, H269, A126 for free energy calculations, and also L122 and L212 for dynamics), and 3-5 water molecules, including 115-151 atoms. The QM region used to probe the quinol oxidation process included the di-iron core ($Fe^{IV}=O^{2-}/Fe^{III}-OH^-$), protein residues near the active site (E123, E162, E213, E266, H165, Y220, R96, L122, E215, T219), as well as the quinol headgroup and 2-3 water molecules, comprising in total *ca.* 168-171 atoms (with an additional water molecule in the open ion-pair model). The QM-MM boundary was modeled by the link-atom scheme, with hydrogen atoms introduced between C_α and C_β positions for protein residues, and between C_{11} and C_{12} positions for the quinol. The QM region was modeled at the B3LYP-D3/def2-SVP(C, O, N, H)/def2-TZVP(Fe) level,[5–8] with the CHARMM36 force field used to describe the MM atoms.[4]

To study the oxygen activation and the quinol oxidation processes, reaction pathway optimizations were performed with restraints on a reaction coordinate, R, driving the system from the reactants to products by a harmonic restraint of 3000 kcal mol^{-1}Å^{-2}. R was sampled in both forward and backward until convergence. For the oxygen activation process, the reaction coordinate of the O-O splitting ($R_{O\text{-}O}$) was defined as the O-O distance, whereas the reaction coordinate for the PCET from Tyr220 to the oxygen (R_{PCET}), was defined as a linear combination of bond breaking and forming distances (see Fig. S5B,E). For the proton transfer between QH_2 and the ferryl ($Fe^{IV}=O^{2-}$), R_1 was defined as the difference of distances between the breaking bond (O5-H of QH_2), and bond forming distances (H-O—Fe, Fig. S11A). For the proton transfer between $QH^\bullet$ and E215, R_2 was defined as the difference in the bond breaking ($QH^\bullet$ O2-H) and bond forming (H-OOC Glu215) distances, connected by a direct hydrogen bond (closed conformation, R_{2a}) or bridged by two water molecules (open ion-pair, R_{2b}) (Fig. S11E,F). During unrestrained QM/MM minimization, 10 Å around the QM region was allowed to relax, whereas for the reaction coordinate optimizations, heavy atoms within the QM region were kept fixed. QM/MM dynamics were performed at T=310 K, with a 1 fs timestep, allowing all atoms to move around 10 Å of the QM region. QM/MM free energy calculations were performed using the umbrella sampling method. The reaction coordinates were divided into 12-25 windows that spanned the full range of R (see Fig. S5, S11). Windows were restrained to their corresponding R values with a harmonic potential using a force constant of 100-500 kcal mol^{-1} Å^{-2} for the proton transfer and PCET reactions, or 500-2000 kcal mol^{-1}Å^{-2} for the O-O splitting, with up to 3 ps sampling/window at T=310 K, resulting in a total of 30-60 ps for each reaction. The free energy profiles were computed using the weighted-histogram analysis method (WHAM), whereas Monte Carlo bootstrap analysis was used to estimate statistical errors.[15] All QM/MM calculations were performed with TURBOMOLE[14] and CHARMM,[16] coupled by a python interface.[17]

Protein expression, purification, and activity measurements

AOX was expressed and purified by slightly modifying a previous protocol[18], by changing the induction time to 3 hours. To this end, His-tagged AOX variants from *T. brucei* were inserted in a pET15b vector, by replacing the signal peptide sequence (residues 1-24) with a 6-His tag and a thrombin cleavage site, and over-expressing the constructs in *E. coli* BL21 cells. Pre-cultured cells were transferred into LB growth medium and 100 μg mL^{-1} ampicillin, until OD_{600} = 0.6 at 37°C. Expression was induced for 3 h at 30°C following addition of 250 μM IPTG, and cells were harvested by centrifugation for 12 min at 6,000 g. Cells pellets were re-suspended in 50 mM Tris-HCl (pH=8), 1 mM $MgSO_4$ with protease inhibitor cocktail (Sigma), and the cells were broken by sonication, followed by harvesting at 6,000 g for 12 min, and membranes were collected by ultra-centrifugation at 165,000 g for 1h 30 min at 4°C. The membranes were resuspended to 6 mg of protein per mL^{-1} using a resuspension buffer with 25 mM Tris-HCl (pH 8), 200 mM $MgSO_4$, 20% (v/v) glycerol. Membrane proteins were then solubilized by adding 1.4% β-OG at 4°C, and stirring for 1 h, after which the

pellet was discarded. Solubilized AOX was loaded on a His-Trap HP column (Cytiva), and the column was washed with 10 CV of buffer A (20 mM Tris-HCl pH 8, 50 mM $MgSO_4$, 150 mM NaCl, 20 mM imidazole, 20% (v/v) glycerol). Elution was performed first using 10 CV of 20% buffer B (20 mM Tris-HCl pH 8, 50 mM $MgSO_4$, 150 mM NaCl, 300 mM imidazole, 0.05% (w/v) DDM, 20% (v/v) glycerol) to wash the non-specific bound proteins, followed by a gradient from 20% to 100% buffer B over 15 CV. A buffer exchange was performed by size-exclusion chromatography in 20 mM MOPS pH 7.5, 150 mM NaCl, 0.05% (w/v) DDM. The iron stoichiometry following the protein purification was assessed in Refs.[19,20] Oxidation of quinol (Q_1H_2) to quinone (Q_1) was measured using 0.1-0.7 µg of WT or mutant AOX in a reaction buffer with 50 mM MOPS at pH 7.5 and 150 mM NaCl. The quinol oxidation was followed optically at 278 nm (ε=15 000 M^{-1} cm^{-1}).

The AOX variants were created by overlapping PCR, by engineering suitable primers (Table S4), which were ordered from Eurogentec. PCRs were performed using the Q5 DNA polymerase. The final step of the directed mutagenesis was performed by the DpnI restriction enzyme to digest the non-mutated DNA template. The final product was transformed into *E. coli* DH5α strain, and introduced substitutions were confirmed by sequencing.

Activity assays were performed by measuring quinol oxidation, using UH5300 Hitachi spectrophotometer, and a quartz cuvette (*d*=1 cm) and monitoring the quinol (Q_1H_2) oxidation to quinone (Q_1) at 278 nm (ε=15 000 M^{-1} cm^{-1}) with 0.1-0.7 µg of WT or mutant AOX, in a reaction buffer with 50 mM MOPS pH 7.5 and 150 mM NaCl. Prior to the quinol assay, Q_1, was solubilized in 50 mM MOPS pH 7.5 and 30% ethanol, and Q_1 was reduced to Q_1H_2 via sonication with zinc powder under an inert atmosphere for *ca. 2*0 min, following removal of the zinc by centrifugation. To avoid the slow self-oxidation of Q_1H_2, aliquots were prepared to be used only once, and treating the kinetics in the same way. The O_2 consumption measurements were initiated by adding 1 mM of AOX. The K_m for Q_1H_2 was determined by a least-square fit to Michaelis-Menten equations (Fig. 4), using GraphPad Prism v. 7.0, GraphPad Software, La Jolla California USA, www.graphpad.com. All reagents were purchased from Sigma-Aldrich if not otherwise stated.

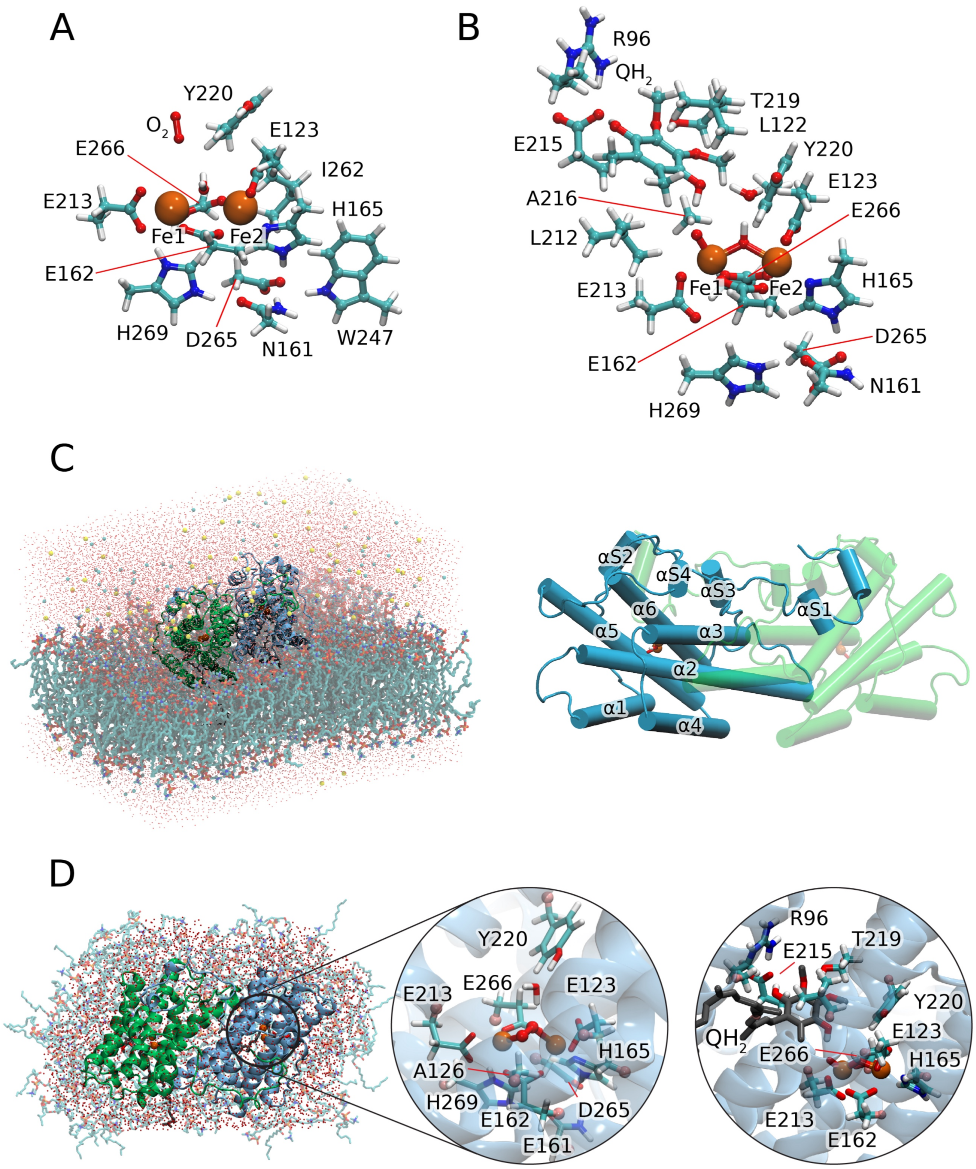

Figure S1. DFT, MD, and QM/MM models. A) DFT models used for probing the oxygen activation and splitting reaction. The models contain the di-iron core, first- and second-sphere protein residues, a coordinating water molecule, O_2, and the catalytically central Tyr220. The models comprise *ca.* 140 atoms, and were constructed based on the coordinates of the x-ray structure of AOX (PDB ID: 3VV9[1]). **B)** DFT models with *ca*. 200 atoms for probing the QH_2 oxidation reaction. The models contain the di-iron core and surrounding residues, the quinol substrate, and residues around the putative proton acceptor site. QH_2 was modeled based on a relaxed structure obtained from the MD simulations. See Table S1 for further technical details. **C)** MD simulation setup of AOX, showing the protein dimer, the POPC lipid membrane, water molecules, and ions. **D)** QM/MM setup, with the system comprising around 40,000 atoms, including the AOX dimer and its surroundings. *Inset*: residues included in the QM region (left: oxygen activation; right: quinol oxidation).

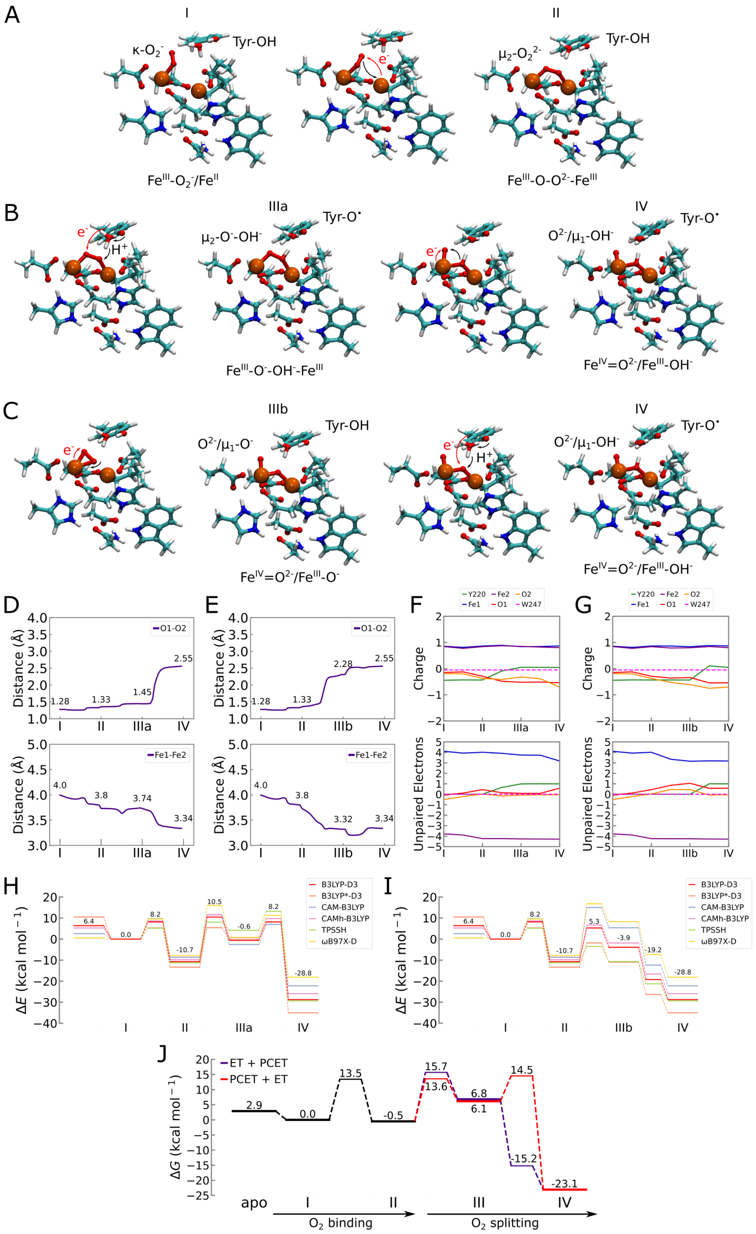

Figure S2. Optimized DFT models for the oxygen splitting reaction. A) Oxygen binding to the active site in I) the end-on (*left*), the transition state (*middle*), and II) side-on (*right*) conformations. **B)** Oxygen splitting by PCET from Tyr220, following a peroxy-intermediate (IIIa), and splitting of the O-OH bond that results in a ferryl state (IV) (PCET+ET pathway). **C)** Oxygen splitting by splitting of the O-O bond (state IIIb), following PCET from Tyr220, which results in a ferryl state (IV) (ET+PCET pathway). **D**, **E)** O1-O2 (top) and Fe1-Fe2 (bottom) distances during the **D**) PCET+ET and **E**) ET+PCET pathways. **F**, **G)** Charge and spin populations during oxygen splitting catalysis (**F**, PCET+ET with PCET from Tyr220 first, **G**, ET+PCET with oxygen-splitting first). **H**, **I)** Benchmarking the energetics of the oxygen splitting reaction (**H**, PCET+ET; **I**, ET+PCET) with different density functionals (B3LYP-D3[5–7], B3LYP*-D3[5–7,21], CAM-B3LYP-D3[7,22], CAMh-B3LYP-D3[7,23], TPSSh-D3[7,24] and ωB97-XD[25]) at the def2-TZVP/ε=4 level.[8–10] **J)** Free energy for the oxygen-splitting reaction based on the DFT models: *apo* state (+2.9 kcal mol^{-1}), followed by I) O_2 binding to Fe1 in the κ^1 mode, II) relaxation to the side-on (μ_2) mode, where dioxygen bridges both Fe atoms. III) Energetics of two competing O_2-splitting pathways: 1) water-mediated PCET from Tyr220 to O_2 followed by splitting of the $(O\text{-}OH)^-$ bond (in red, PCET+ET), and 2) O-O bond splitting followed by PCET from Tyr220 (in purple, ET+PCET). Both pathways result in (IV) a $Fe^{IV}=O/Fe^{III}\text{-}OH^-$ state and tyrosyl radical.

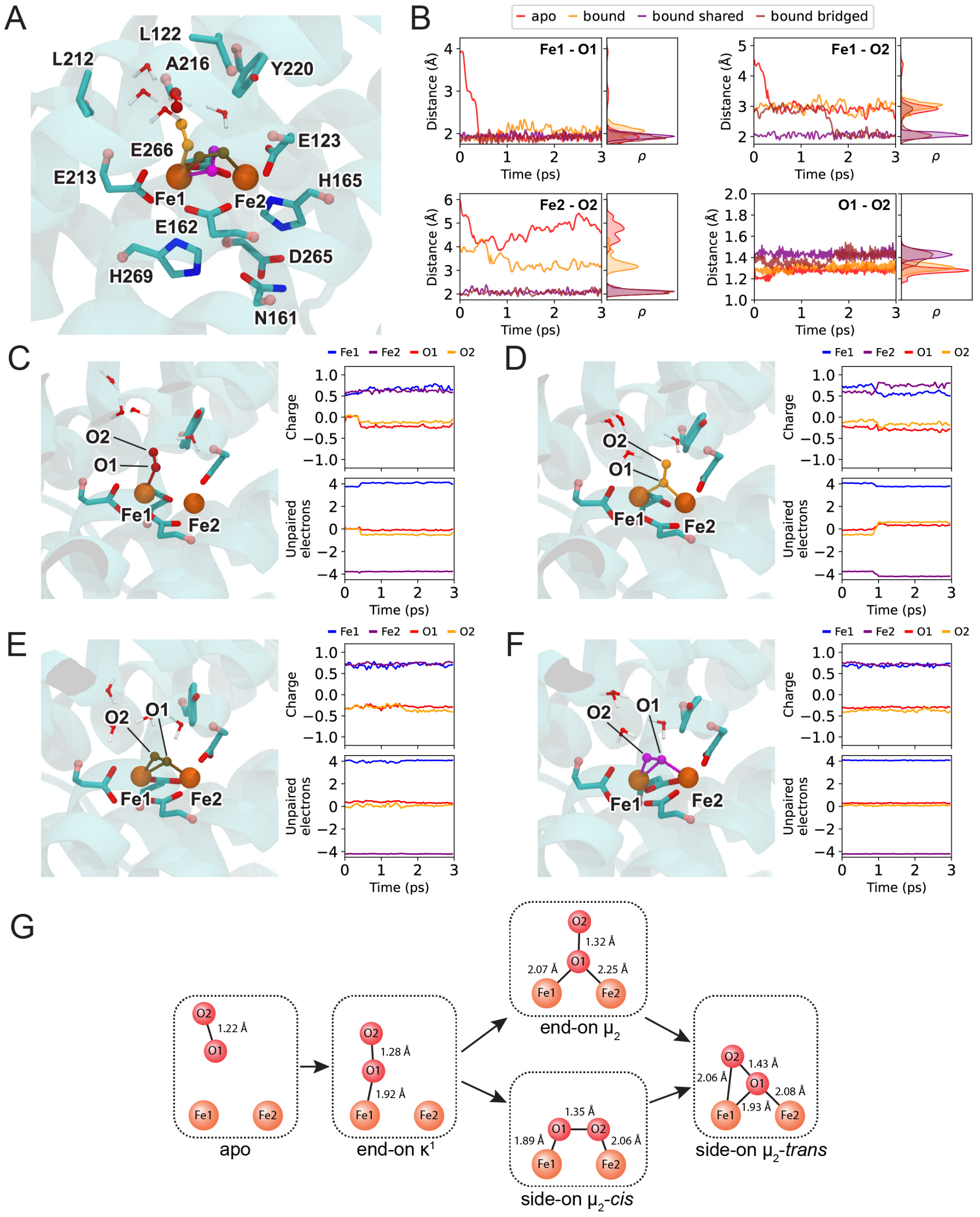

Figure S3. Dynamics of the oxygen binding modes at the QM/MM level. A) The QM/MM model, showing protein residues included in the QM region, and the different initial oxygen positions studied (red: *apo*; yellow: *end-on* (κ^1) mode; purple: *side-on* (μ_2)-mode (bridged); brown: *side-on* (μ_2)-mode (shared)). **B)** Distance analysis between the atoms Fe1, Fe2, O1, and O2. **C-F)** Representative snapshots of the binding mode (*left*) from QM/MM dynamics simulations **C)** *end-on* (κ^1) mode; **D)** *side-on* (μ_2)-mode (bridged); **E)** *side-on* (μ_2-*cis*)-mode (shared); **F)** *side-on* (μ_2-*trans*)-mode (shared). Charge and spin population analysis (*right*). **G)** Schematic representation of the oxygen binding modes and the respective bond lengths.

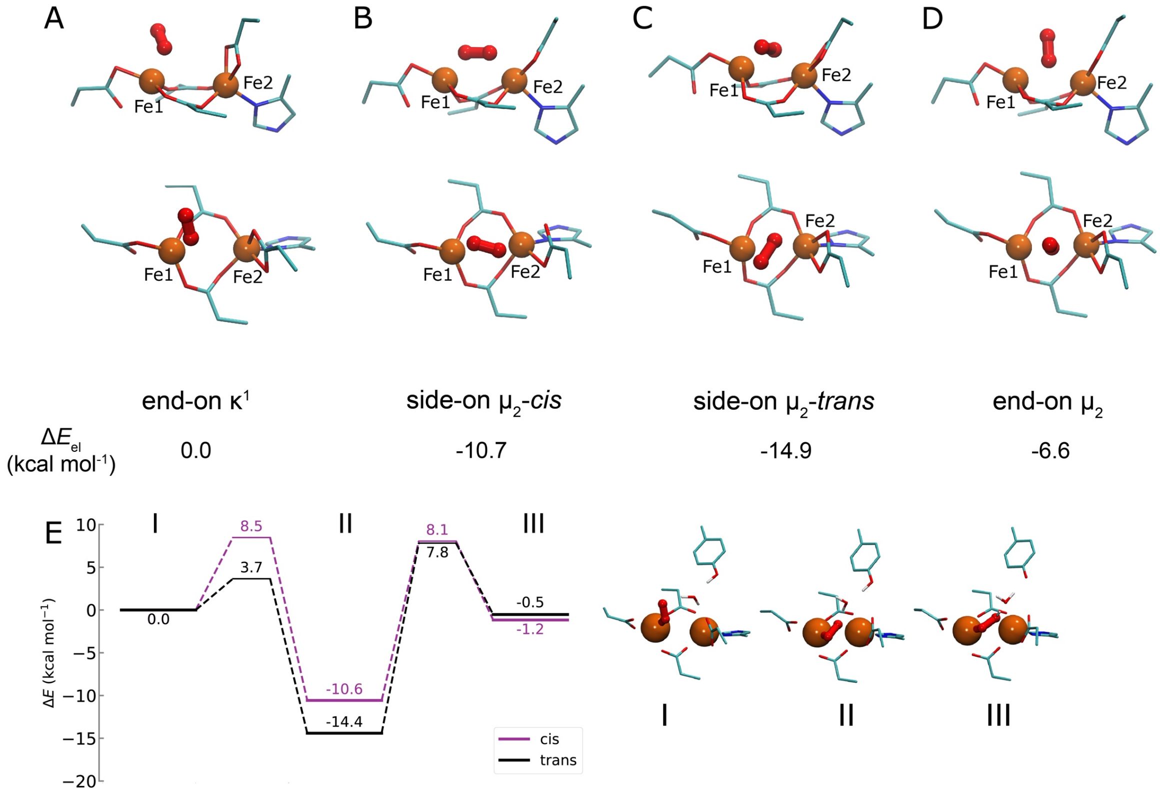

Figure S4. Exploration of different O_2-binding modes at the di-iron site of AOX from DFT models. Figures on the top / bottom represent a side / top view of the active site, respectively. **A)** The *end-on* κ[1] binding mode, with one oxygen binding to Fe1. **B, C)** The *side*-on μ_2 mode in **B)** *cis*- and **C)** *trans* configurations, with both oxygens bridging the Fe1 and Fe2. **D)** The *end-on* μ_2 mode, with only one oxygen atom bridging both irons. The figure reports relative electronic energies (ΔE_{el}) without thermodynamic corrections in kcal mol^{-1} with respect to the *end-on* κ[1] binding mode. **E)** Energetics of the end-on κ[1] mode (I) to side-on μ_2 mode (purple: *cis*; black: *trans*), and the subsequent PCET from Tyr220 to the oxygen in the *cis* and *trans* conformations. Structures of the *side*-on μ_2 *trans* mode are shown on the right.

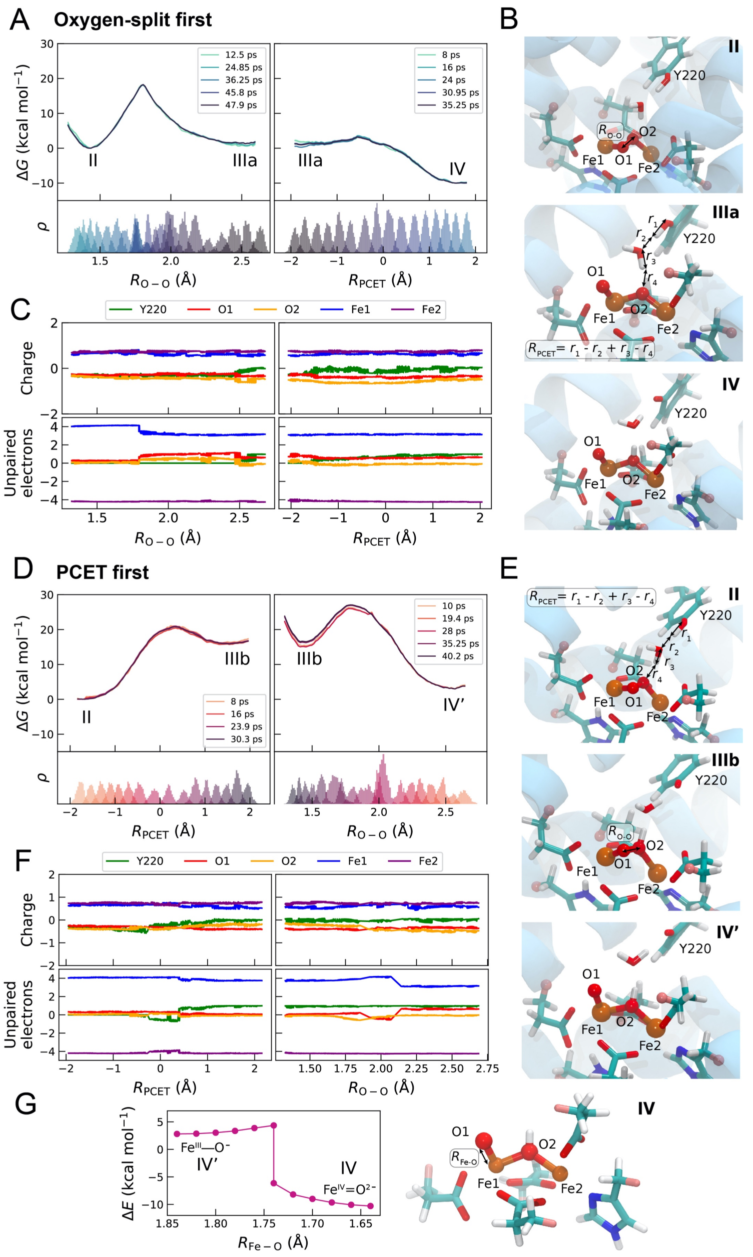

Figure S5. Energetics of the oxygen activation process at the QM/MM level. A, D) QM/MM free energy profile for the oxygen activation and splitting process along the two proposed pathways. The plots show the convergence of the free energy with different levels of sampling. Statistical (WHAM) errors derived from bootstrapping analysis are shown in the *top* panel, and the umbrella sampling windows in the *bottom* panel. **A)** QM/MM free energy profile for pathway 1 (O-O split followed by PCET; *left*: O-O bond splitting, $R_{O\text{-}O}$; *right:* PCET from Tyr220, R_{PCET}). **B)** *Top:* starting structure with the O-O *side-on* (μ_2)-mode, showing the reaction coordinate $R_{O\text{-}O}$ for the O-O bond splitting, defined as the O1-O2 distance; *center*: intermediate state resulting from the O-O bond splitting. The reaction coordinate for the subsequent PCET step, R_{PCET}, is defined as a linear combination of bond-breaking and bond-forming distances; *bottom*: the resulting $Fe^{IV}{=}O^{2-}$/Fe^{III}-OH^-/Tyr-$O^{\bullet}$ state. **C)** Charge (top) and spin (bottom) population analysis along the reaction coordinates. **D)** QM/MM free energy profile for pathway 2 (PCET followed by O-O split; *left*: PCET from Tyr220, R_{PCET}; *right:* O-O bond splitting, $R_{O\text{-}O}$). **E)** *Top:* starting structure with the O-O *side-on* (μ_2)-mode, showing the reaction coordinate for the PCET step (R_{PCET}, linear combination of bond-breaking/forming distances) from Tyr220 to the O-O moiety, mediated by a water molecule; *center*: intermediate O-OH/ Tyr-$O^{\bullet}$ state, showing the $R_{O\text{-}O}$ coordinate for the subsequent O-O splitting; *bottom*: transient Fe^{III}-O^-/Fe^{III}-OH^-/Tyr-$O^{\bullet}$ state, which relaxes into the $Fe^{IV}{=}O^{2-}$/Fe^{III}-OH^-/Tyr-$O^{\bullet}$ state by elongation of the Fe-O bond (see **G**). **F)** Charge (top) and spin (bottom) population analysis along the reaction coordinates. **G)** Relaxation of the transient Fe^{III}-O^- into the high-valent $Fe^{IV}{=}O^{2-}$ state by shortening of the Fe-O bond.

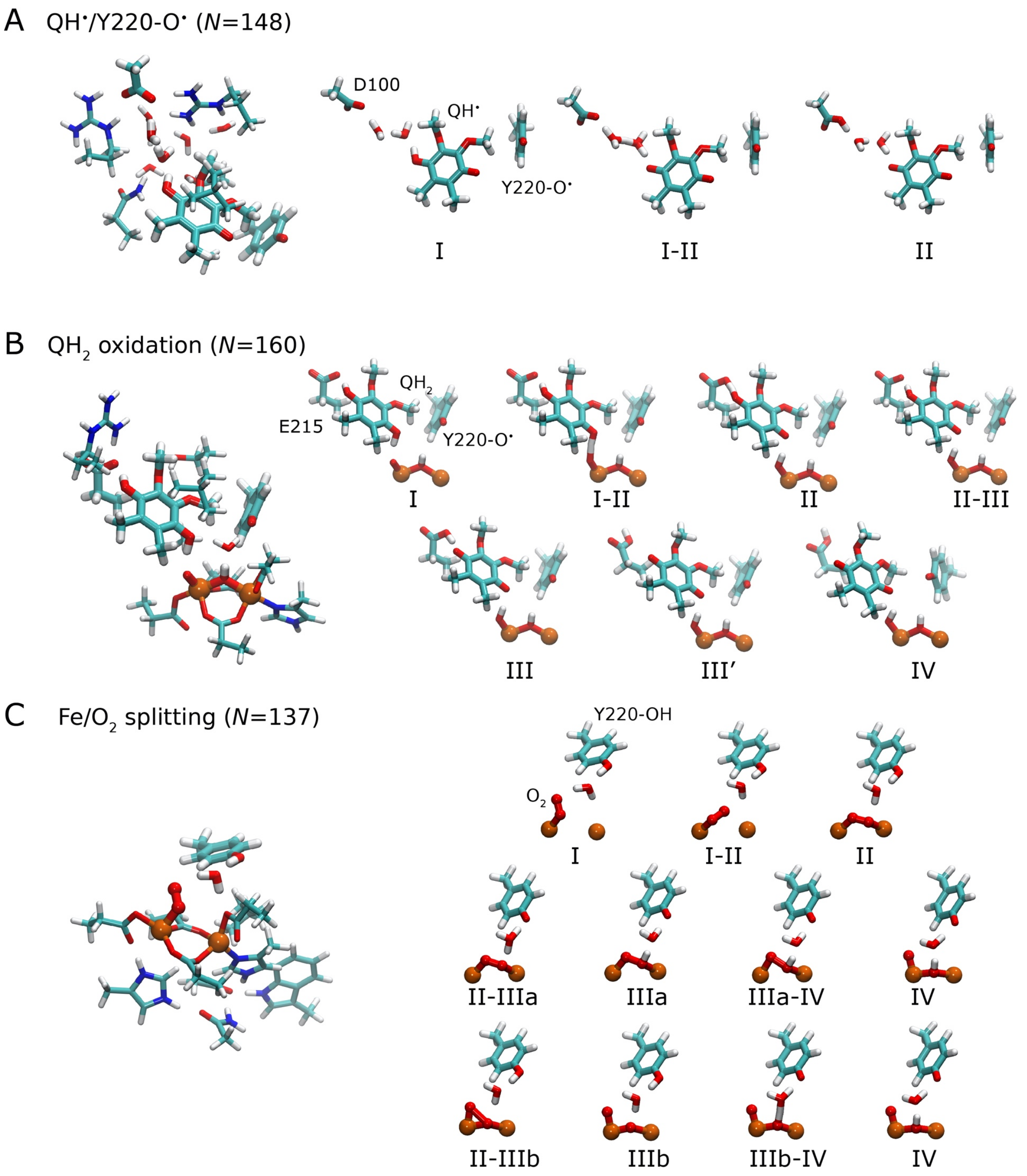

Figure S6. QM models used for the benchmarking calculations. A) Model system of the $QH^{\bullet}$ and Tyr220-$O^{\bullet}$ radical species. *Left*: full model system with N = 148 atoms; *right*: reaction intermediates, showing the atoms and residues involved in the reaction pathway of the proton transfer from $QH^{\bullet}$ to Asp100. **B)** Model system of the QH_2 oxidation reaction. *Left:* benchmarking model with N = 160 atoms (*left*) that was built based on the larger DFT models of the QH_2 oxidation process (N=203 atoms). *Right*: reaction intermediates, showing QH_2, Tyr220, Glu215, and the di-iron core. I-II: PCET from QH_2 to Fe^{IV}=O^{2-}; II-III: pT from $QH^{\bullet}$ to Glu215; IV: eT from $Q^{\bullet}$ to Tyr220-$O^{\bullet}$. **C)** Model system for the oxygen splitting reaction. *Left*: full model (N=137 atoms). *Right*: reaction intermediates, showing Tyr220, O_2, the di-iron core, and an intervening water molecule. I-II: oxygen binding from *end-on* mode to *side-on* mode; II-IIIa: PCET from Tyr220 followed by O-O bond splitting; II-IIIb: O-O splitting, followed by PCET from Tyr220. Both states IIIa and IIIb result in state IV along the explored reaction pathways (see main text).

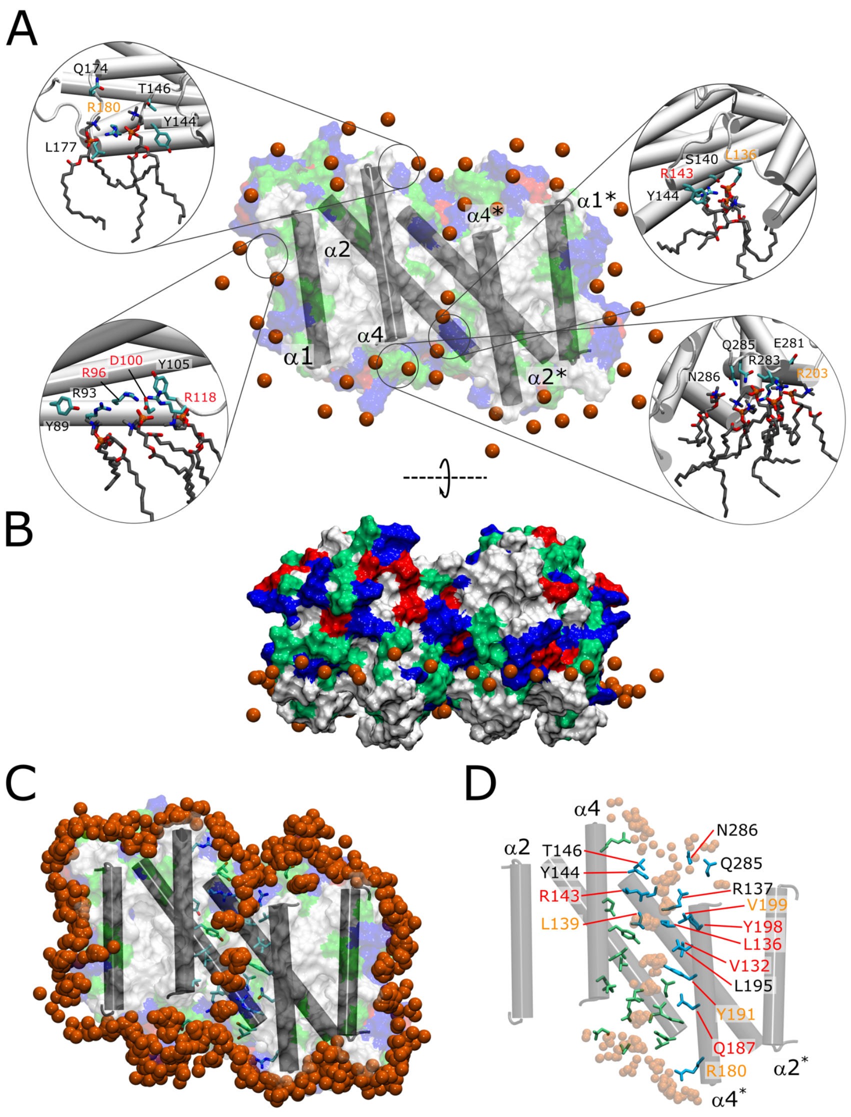

Figure S7. AOX-membrane interactions. A) Bottom view of the interaction between the dimeric AOX and lipid headgroups (orange spheres) from a snapshot of a 0.5 μs MD simulation (simulation S3, Table S2). The protein is shown in surface representation with hydrophobic (light grey), polar (green), positive (blue), and negative (red) residues. Insets show protein residues interacting with lipids at specific sites: Fully conserved residues (labels in red); partially conserved residues (in orange); non-conserved residues (in black). **B)** Side view of the protein-lipid interactions. **C)** Ensemble-averaged protein-lipid interactions (orange spheres) from MD simulation (simulation S3, Table S2). **D)** Lipid headgroup - protein sidechain interactions (chain A in cyan, chain B in green) within the cavity formed by the helices α4, α2, α4*, and α2*. Fully conserved residues (labels in red); partially conserved residues (in orange); non-conserved residues (in black).

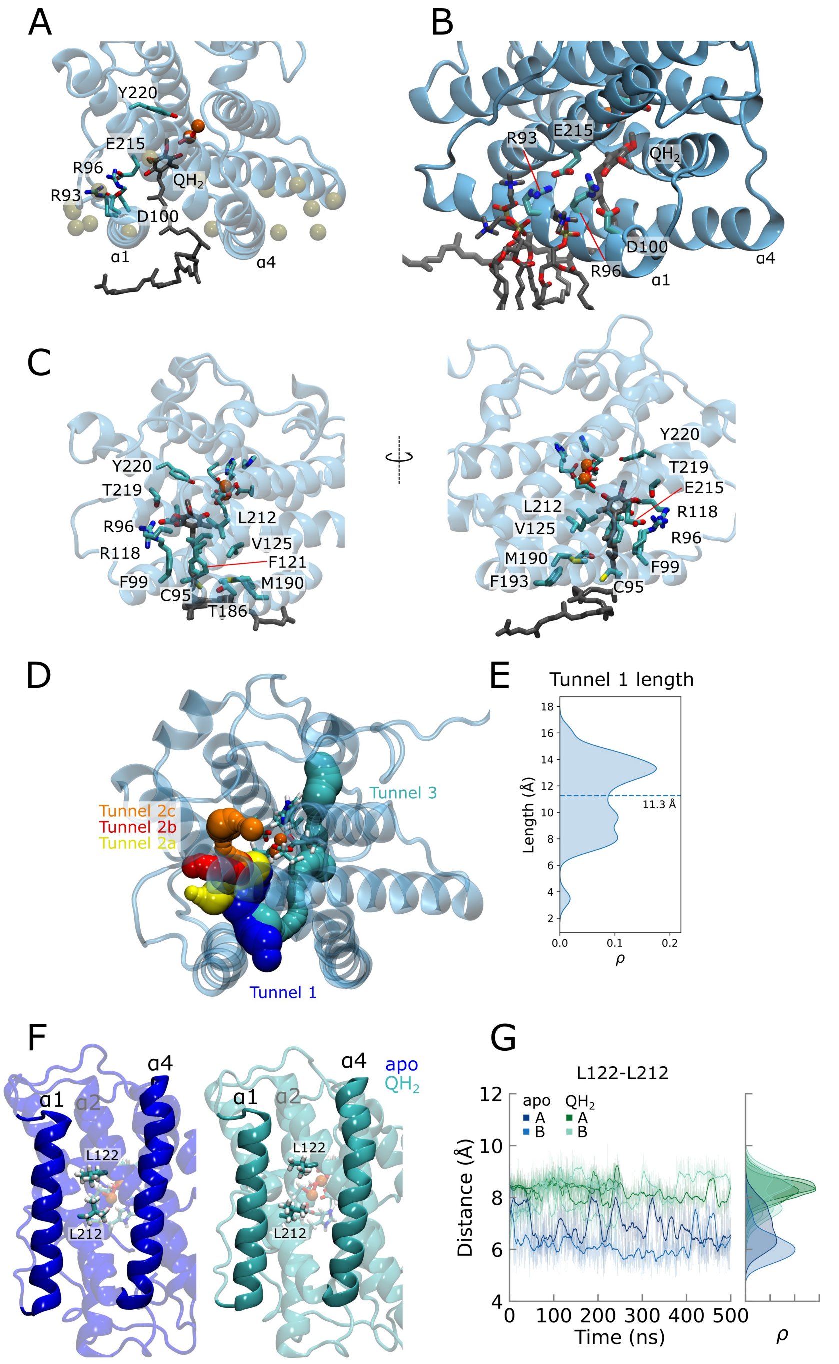

Figure S8. Quinol binding cavity. A) Snapshot from MD simulations showing the interaction of lipid headgroups (tan spheres) with helices α1 and α4. The quinone binding site is surrounded by the conserved residues Glu215, Arg96, Asp100, and Tyr220. **B)** Arg93 and Arg96, located on helix α1, interact with lipid molecules (in dark grey). **C)** The quinol cavity is mostly hydrophobic with key polar/charged residues (Arg96, Asp100, Arg118, Glu215, Thr219) located near the active site. The entrance is formed by Cys95, Met190, and Phe193. **D)** Three main tunnel clusters could be identified during the MD simulation. Tunnel 1 accommodates the quinol tail, while tunnels 2 and 3 are occupied by water molecules. **E)** The length of tunnel 1 during the MD simulation with quinol (S1) with an average length of 11.3 Å. **F)** Snapshots from MD simulations with QH_2 or no substrate (*apo* state) show opening and closing of the Q-cavity, respectively. **G)** Distance between the gating residues, Leu122 and Leu212, from MD simulations of the QH_2 state (simulations S1, S2, S3) and the *apo* state (simulation S12, see Table S2).

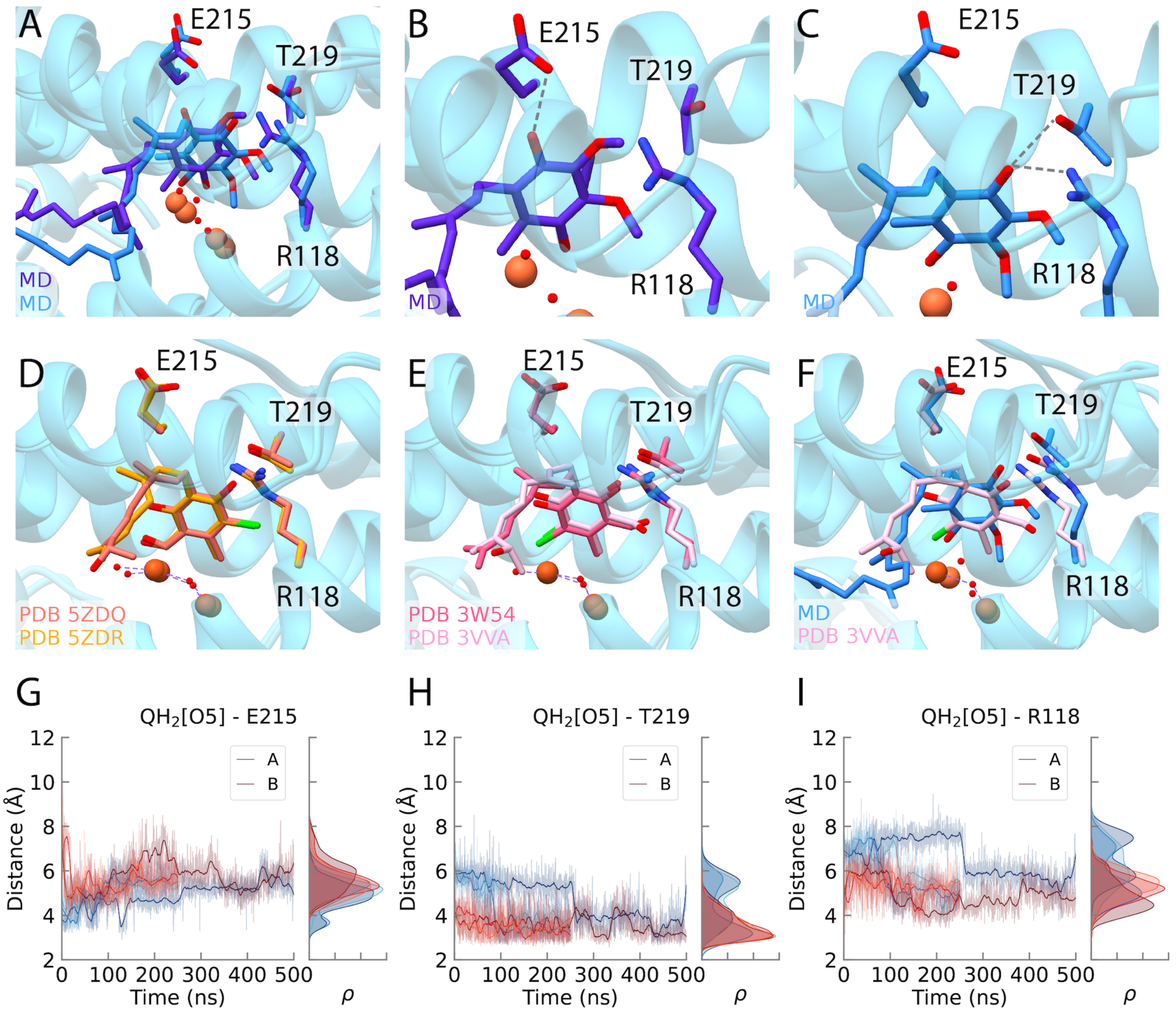

Figure S9. Structural comparison of inhibitor-bound crystal structures and MD simulations. A) Dynamics of QH_2 in the active site from MD simulations (simulations S1-S2, Table S2). The QH_2 interaction with **B**) Glu215 and **C**) Thr219 from MD simulations from two MD snapshots. **D-E)** Crystal structures of inhibitor-bound AOX with ascofuranone (PDB ID: 5ZDQ[26], 3VVA[1]) and colletochlorin B (PDB ID: 5ZDR[26], 3W54[1]), showing different orientations of the inhibitor aromatic ring, interacting with Thr219/Arg118. **F)** Superposition of substrate from MD simulation (simulation S1) and inhibitor-bound crystal structure (PDB ID: 3VVA). See Figure S9 for distances between **G)** the quinol/Glu215, **H)** the quinol/Thr219, and **I)** the quinol/Arg118 during MD simulations (monomer A, blue; monomer B, red; simulations S1, S2, see Table S2).

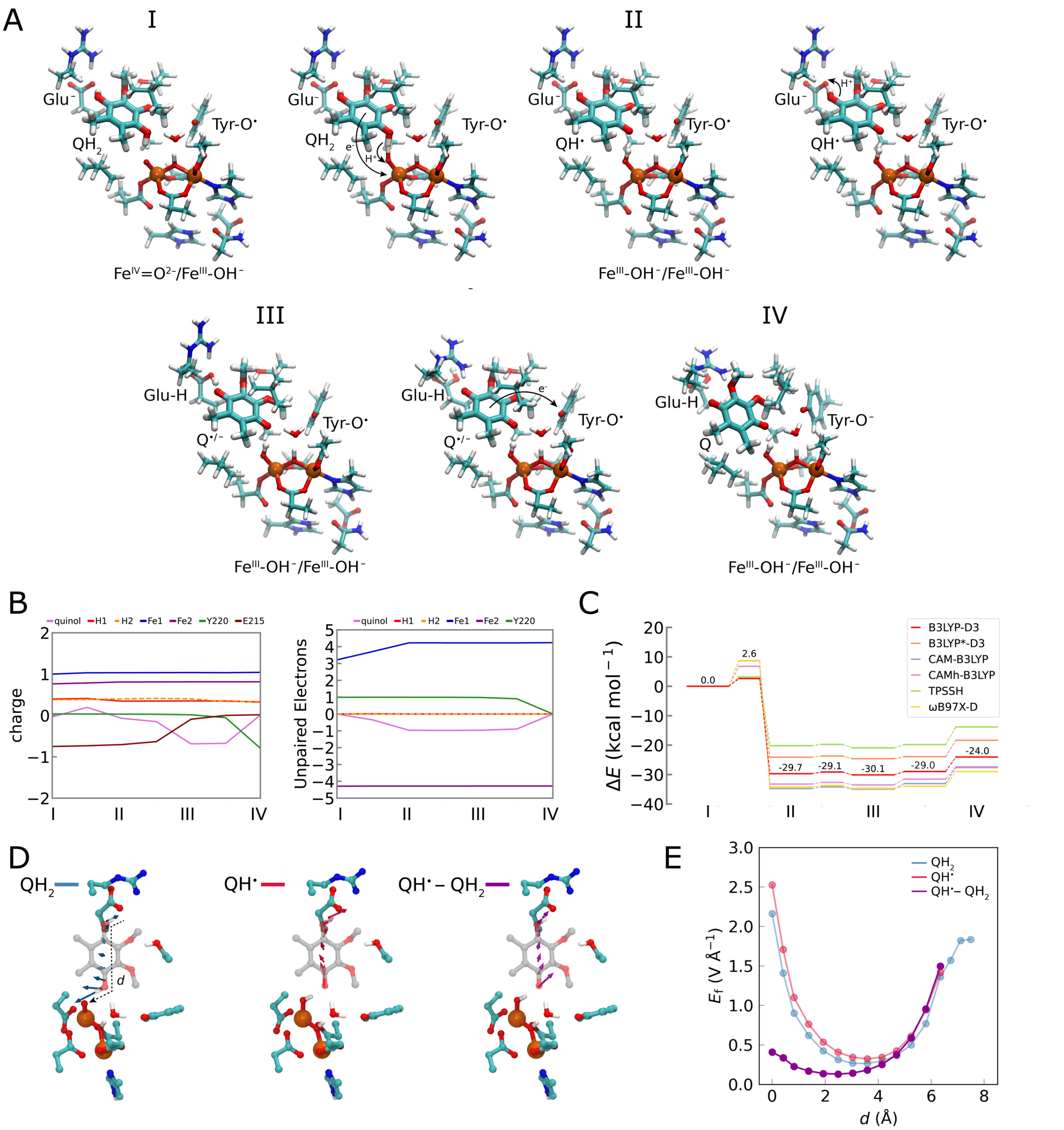

Figure S10. Optimized DFT models for the first quinol oxidation reaction. A) DFT structures correspond to I) $QH_2/Fe^{IV}=O^{2-}/Fe^{III}\text{-}OH^-/Tyr\text{-}O^{\bullet}/Glu^-$; II) $QH^{\bullet}/Fe^{III}\text{-}OH^-/Fe^{III}\text{-}OH^-/Tyr\text{-}O^{\bullet}/Glu^-$; III) $Q^-/Fe^{III}\text{-}OH^-/Fe^{III}\text{-}OH^-/Tyr\text{-}O^{\bullet}/Glu\text{-}H$; IV) $Q/Fe^{III}\text{-}OH^-/Fe^{III}\text{-}OH^-/Tyr\text{-}O^-/Glu\text{-}H$. **B)** Population analysis (Mulliken charge, *left*; spin, *right*) for the quinol oxidation reaction at the DFT level. **C**) Benchmarking the energetics of the QH_2 oxidation reaction with different density functionals (B3LYP-D3[5–7], B3LYP*-D3[5–7,21], CAM-B3LYP-D3[7,22], CAMh-B3LYP-D3[7,23], TPSSh-D3[7,24], and ωB97-XD[25]) at the def2-TZVP/ε=4 level.[8–10] **D)** Electric field vectors along the substrate cavity during the quinol oxidation reaction in state I ($QH_2/Fe^{IV}=O^{2-}/Fe^{III}\text{-}OH^-$) indicated with blue vectors (*left*); state II ($QH^{\bullet}/ Fe^{III}\text{-}OH^-/Fe^{III}\text{-}OH^-$) indicated with red vectors (*middle*); and the electric field difference vector (state II – state I) indicated with purple vectors (*right*). **E)** Electric field strength along the substrate cavity.

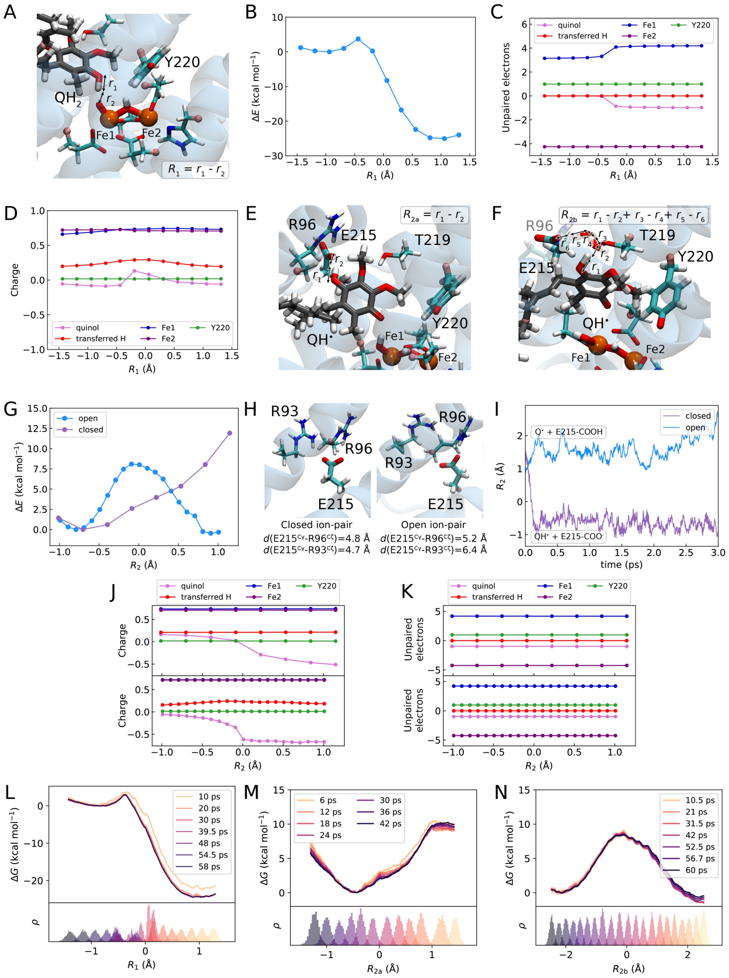

Figure S11. Energetics of the quinol oxidation process at the QM/MM level. A) Starting structure with the QH_2 hydroxyl interacting with $Fe^{IV}=O^{2-}$. The reaction coordinate for the PCET process ($R_1 = r_1$-r_2) was defined as the difference of QH_2-OH bond distance (r_1) and the distance between the transferred H to the $Fe^{IV}=O^{2-}$ (r_2). **B)** Energy profile of the PCET from QH_2 to $Fe^{IV}=O^{2-}$ along the reaction coordinate R_1. **C)** Spin and **D)** charge population along R_1 support PCET from QH_2 to $Fe^{IV}=O^{2-}$, with the electron transfer taking place near the transition state (R_1 = 0). **E, F)** The hydroxyl of the $QH^{\bullet}$ species interacts **E)** directly or **F)** via bridging water molecules with Glu215. **E)** Glu215 in a closed ion-pair conformation with nearby arginines. **F)** The open ion-pair conformation (see panel **H**). **E, F)** The reaction coordinate for the second proton transfer steps, R_2, defined as a linear combination of bond breaking (r_2) and bond forming (r_1) distances between $QH^{\bullet}$ and Glu215. Reaction coordinates for the direct (R_{2a}) and the water-mediated (R_{2b}) process are shown in **E)** and **F)**. **G)** Energy profile of the proton transfer from $QH^{\bullet}$ to Glu215 with a closed ion-pair (in purple) and open ion-pair (in blue). The Glu215-Arg93/96 ion-pair conformations are shown in panel **H**. R_2 was normalized to the [-1 Å,1 Å] range for comparison of the direct and water-mediated reactions. **H)** The Glu215-Arg93/96 ion-pairs in the closed (*left*) and open (*right*) conformations. The distances correspond to the structures prior to the proton transfer reaction, with $QH^{\bullet}$ and anionic Glu215. **I**) Spontaneous back-proton transfer from Glu215 to $Q^{\bullet/-}$ in the closed ion-pair conformation in QM/MM MD simulations, while the proton remains on Glu215 in the open ion-pair conformation. **J)** Spin and **K)** charge population along the second proton transfer step ($QH^{\bullet} \rightarrow$ Glu215). Closed ion-pair conformation (top panels); open ion-pair conformation (bottom panels). The electron remains on $Q^{\bullet}$, whereas partial dissociation of $Q^{\bullet}$ and reorganization of the binding site induce electron transfer from $Q^{\bullet}$ to Tyr220 (see Figure S10). **L-M)** QM/MM free energy profiles of the quinol oxidation process, showing convergence with increasing sampling. Statistical (WHAM) errors derived from bootstrapping analysis in the *top* panel, and the umbrella sampling windows in the *bottom* panel. **L)** QM/MM free energy profile for the PCET reaction from QH_2 to $Fe^{IV}=O^{2-}$, resulting in $QH^{\bullet}$/ Fe^{III}-OH species. **M)** QM/MM free energy profile for the proton transfer from $QH^{\bullet}$ to Glu215 in the closed ion-pair conformation. **N)** QM/MM free energy profile for the proton transfer from $QH^{\bullet}$ to Glu215 in the open ion-pair conformation.

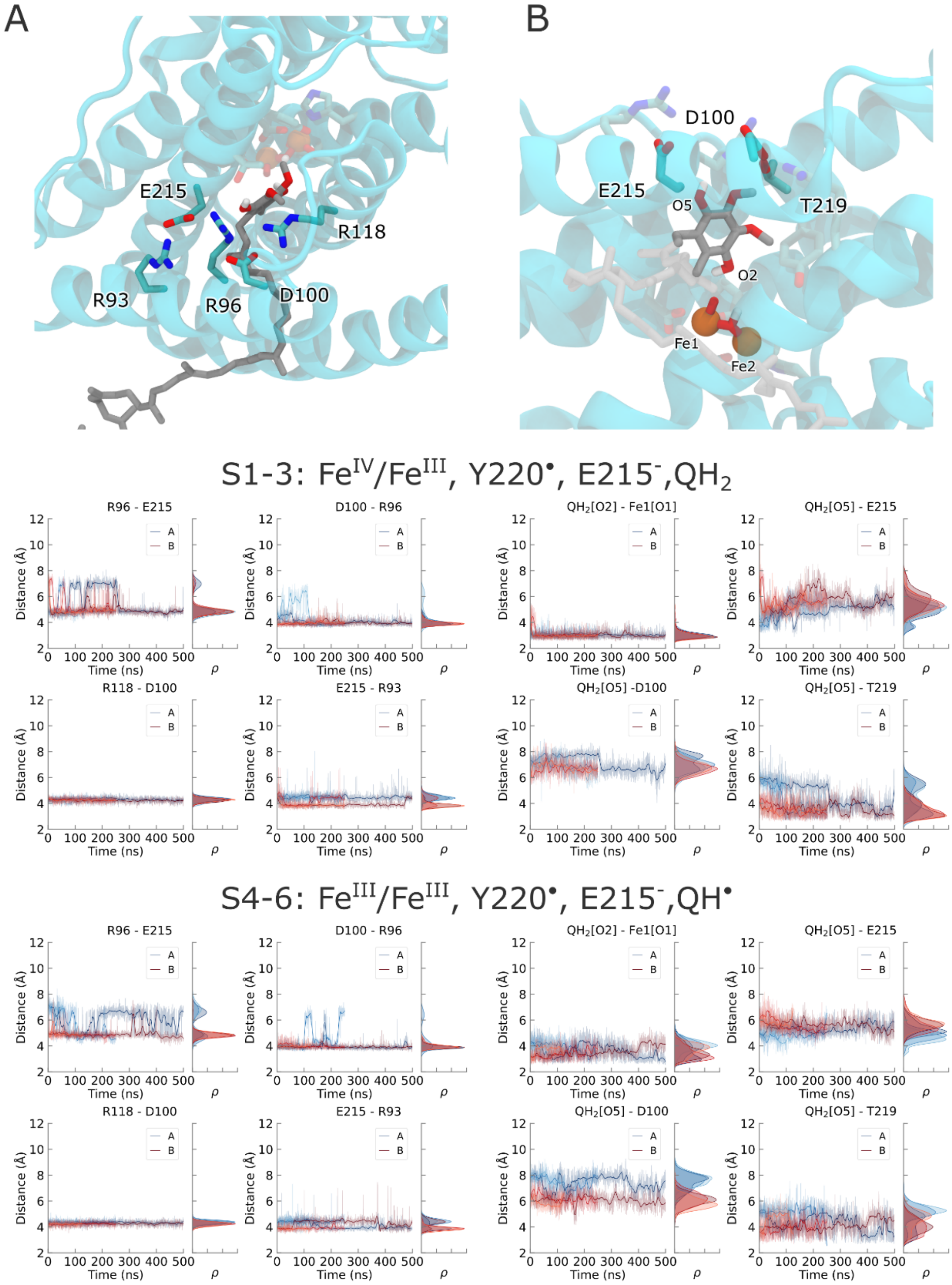

Figure S12. Analysis of distances from MD simulations in WT-AOX. Blue and red trances indicate A and B chains of the AOX dimer, respectively, and replicas are shown in light shade of blue/red (see Table S2). **A)** Snapshot from simulation S1 illustrating the ion-pair network formed by the residues R93, E215, R96, D100, and R118. Distances from individual simulations are shown below. **B)** Snapshot form simulation S1, showing the main QH_2 binding-mode and its interaction with residues E215, D100, and T219, as well as with the ferryl oxygen (Fe1[O1]). Distances from individual simulations are shown below.

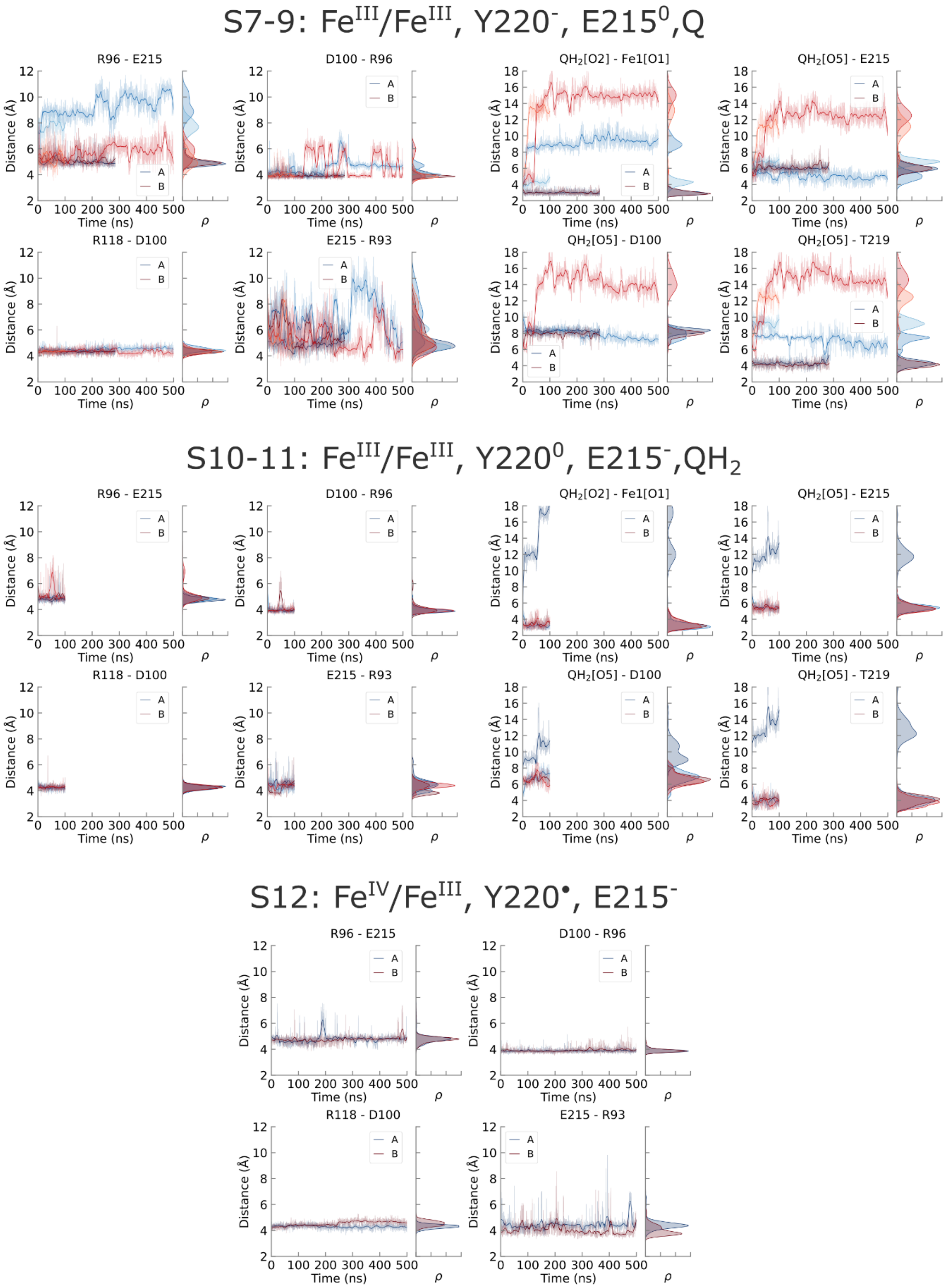

Figure S12 (*contd*). Analysis of distances from MD simulations in WT AOX. Blue and red trances indicate A and B chains of the AOX dimer, respectively, and replicas are shown in light shade of blue/red (see Table S2). **A)** Snapshot from simulation S1 illustrating the ion-pair network formed by the residues R93, E215, R96, D100, and R118. Distances from individual simulations are shown below. **B)** Snapshot form simulation S1, showing the main QH_2 binding-mode and its interaction with residues E215, D100, and T219, as well as with the ferryl oxygen (Fe1[O1]). Distances from individual simulations are shown below.

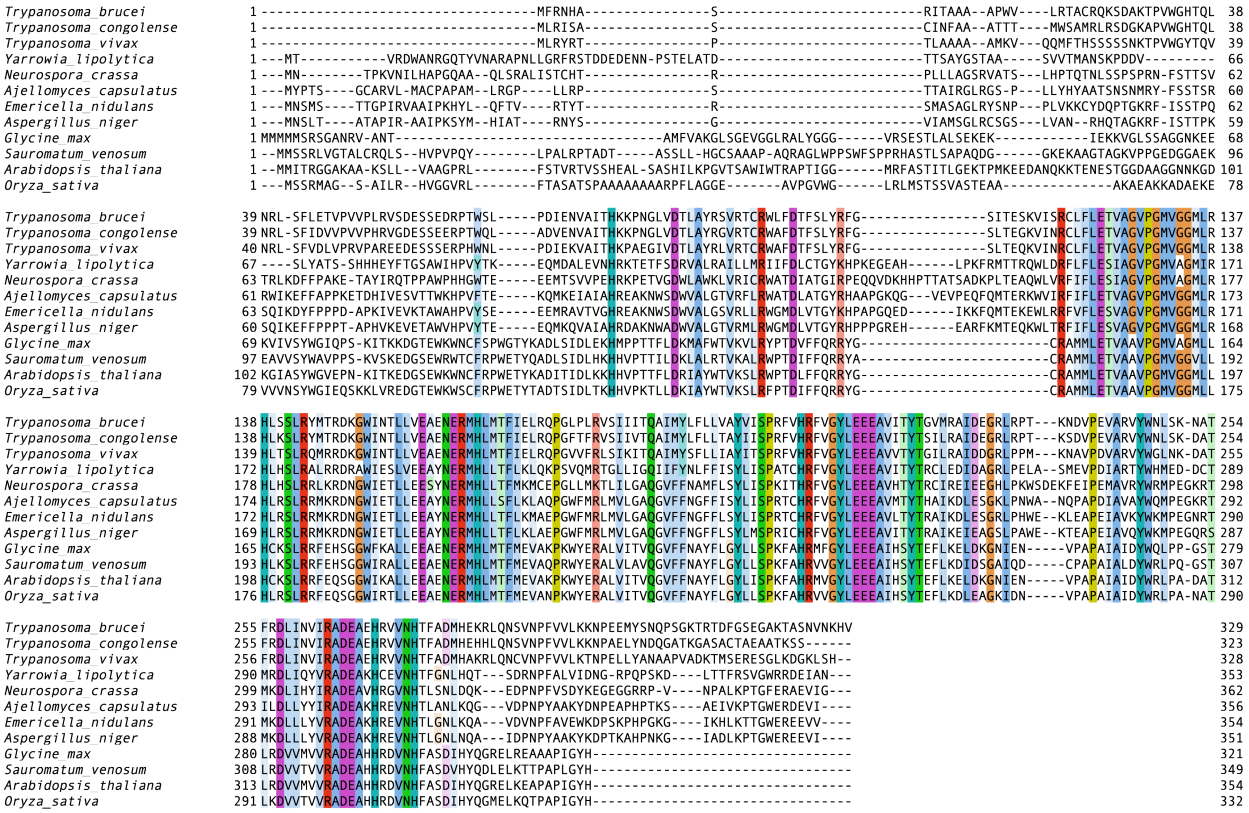

Figure S13. **Multiple sequence alignment** (MSA) of AOX from *Trypanosoma* species, yeast, fungi, and plants. Conserved residues (up to 90% conservation) are highlighted with different colors, using the ClustalX coloring scheme. The MSA was performed with ClustalΩ.[27]

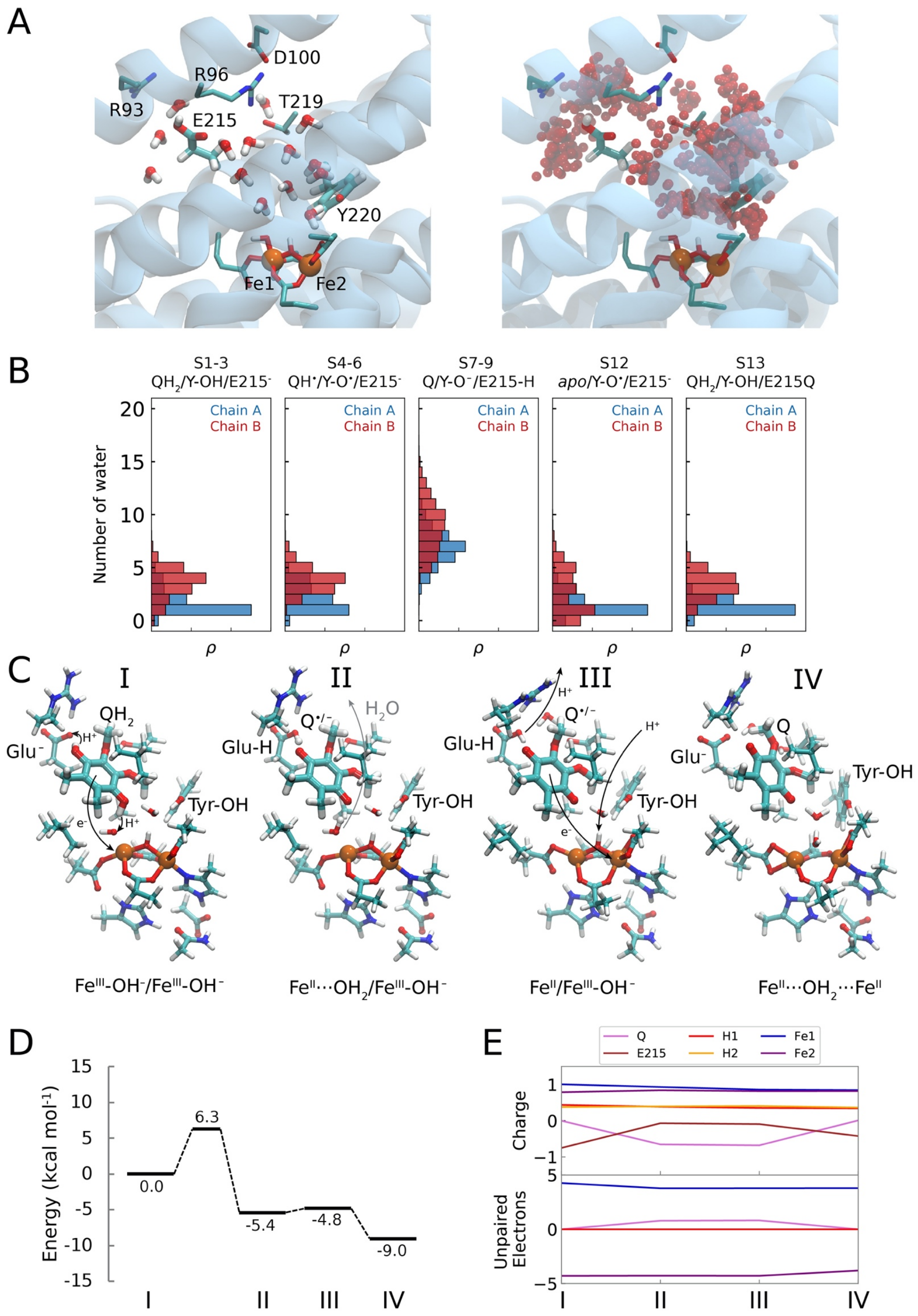

Figure S14. Hydration dynamics and final steps of the reaction cycle. A) Hydration dynamics in the active site cavity after the first quinol oxidation step. After quinone dissociation, the cavity hydrates, which could allow for proton transfer between the protonated Glu215 and the anionic Tyr220. *Left*: representative MD snapshot, showing water connectivity to Tyr220. *Right*: ensemble average of the hydration state over 500 ns MD simulation. **B)** Water count in the active site cavity in different states for the WT AOX: $QH_2/Fe^{IV}{=}O^{2-}/Fe^{III}\text{-}OH^-/Tyr\text{-}O^{\bullet}/Glu^-$ (simulations S1-3); $QH^{\bullet}/Fe^{III}\text{-}OH^-/Fe^{III}\text{-}OH^-/Tyr\text{-}O^{\bullet}/Glu^-$ (S4-6); $Q/Fe^{III}\text{-}OH^-/Fe^{III}\text{-}OH^-/Tyr\text{-}O^-/Glu\text{-}H$ (S7-9); *apo*/$Fe^{IV}{=}O^{2-}/Fe^{III}\text{-}OH^-/Tyr\text{-}O^{\bullet}/Glu^-$ (S12); and for the E215Q-AOX variant: $QH_2/Fe^{IV}{=}O^{2-}/Fe^{III}\text{-}OH^-/Tyr\text{-}O^{\bullet}/Glu^-$ (S13). See Table S2 for list of MD simulations. Water molecules up to 6 Å of the di-iron core and the Tyr220 oxygen are shown. **C)** Optimized DFT models for the second quinol oxidation reaction, with structures corresponding to: I) $QH_2/Fe^{III}\text{-}OH^-/Fe^{III}\text{-}OH^-/Tyr\text{-}OH/Glu^-$; II) $Q^{\bullet/-}/Fe^{II}\cdots OH_2/Fe^{III}\text{-}OH^-/Tyr\text{-}OH/Glu\text{-}H$; III) $Q^{\bullet/-}/Fe^{II}/Fe^{III}\text{-}OH\text{-}/Tyr\text{-}OH/Glu\text{-}H$; IV) $Q/Fe^{II}\cdots OH_2\cdots Fe^{II}/Tyr\text{-}OH/Glu\text{-}H$. **D)** Energetics of the second QH_2 oxidation process. **E)** Spin and charge analysis along the reaction pathway.

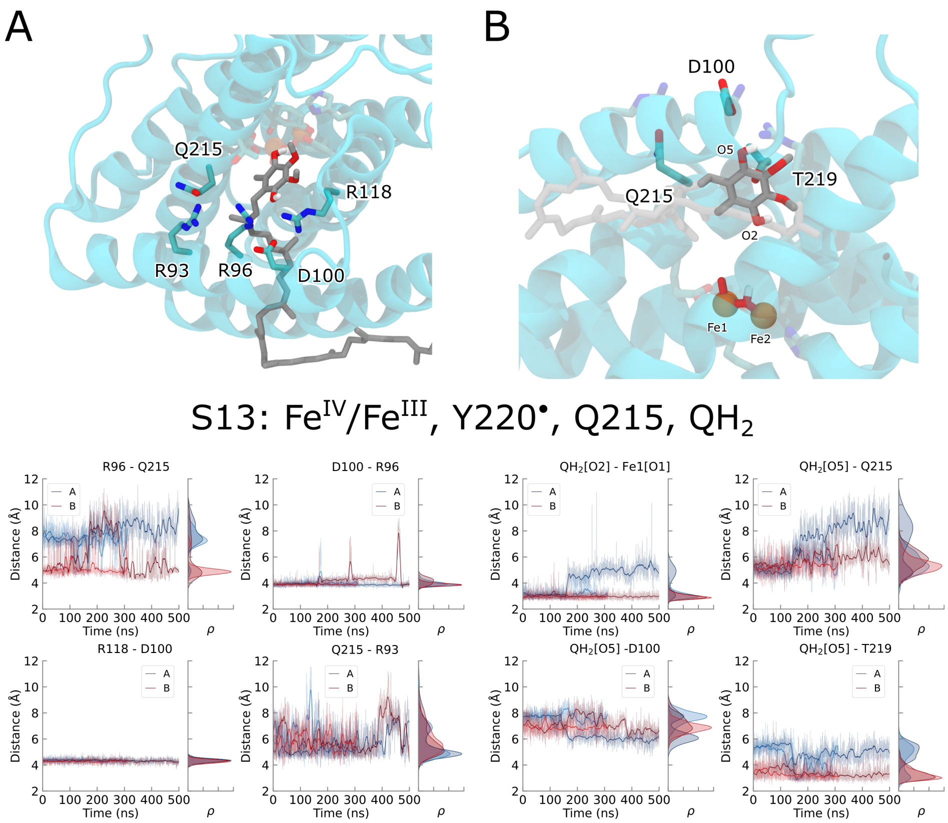

Figure S15. Analysis of distances from MD simulations in the E215Q-AOX variant. Blue and red trances indicate A and B chains of the AOX dimer, respectively, and replicas are shown in light shade of blue/red (see Table S2). **A)** Snapshot from simulation S13 illustrating the ion-pair network formed by the residues R93, Q215, R96, D100, and R118. Distances from individual simulations are shown below. **B)** Snapshot form simulation S13, showing the QH$_2$ binding-mode in the mutant, as well as with the ferryl oxygen (Fe1[O1]). Distances from individual simulations are shown below.

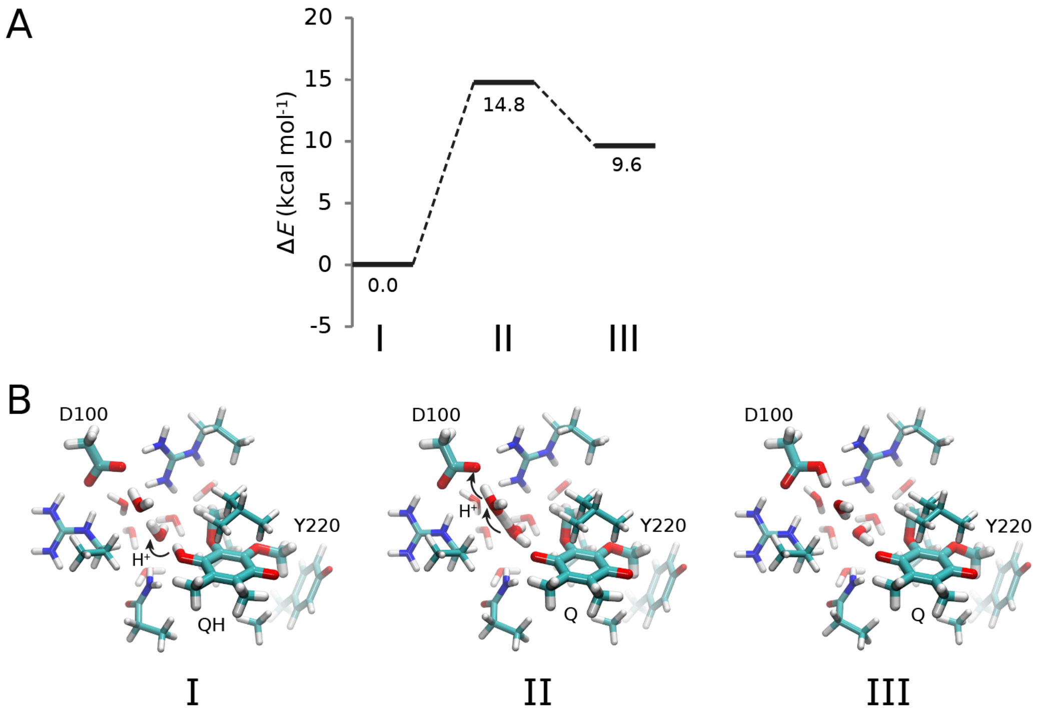

Figure S16. Energetics of proton transfer in the E215Q mutant. A) Energy profile for the proton transfer reaction from QH• to Asp100 at the DFT level. **B**) Optimized structures of the I) reactant state, II) transition state, and III) product state.

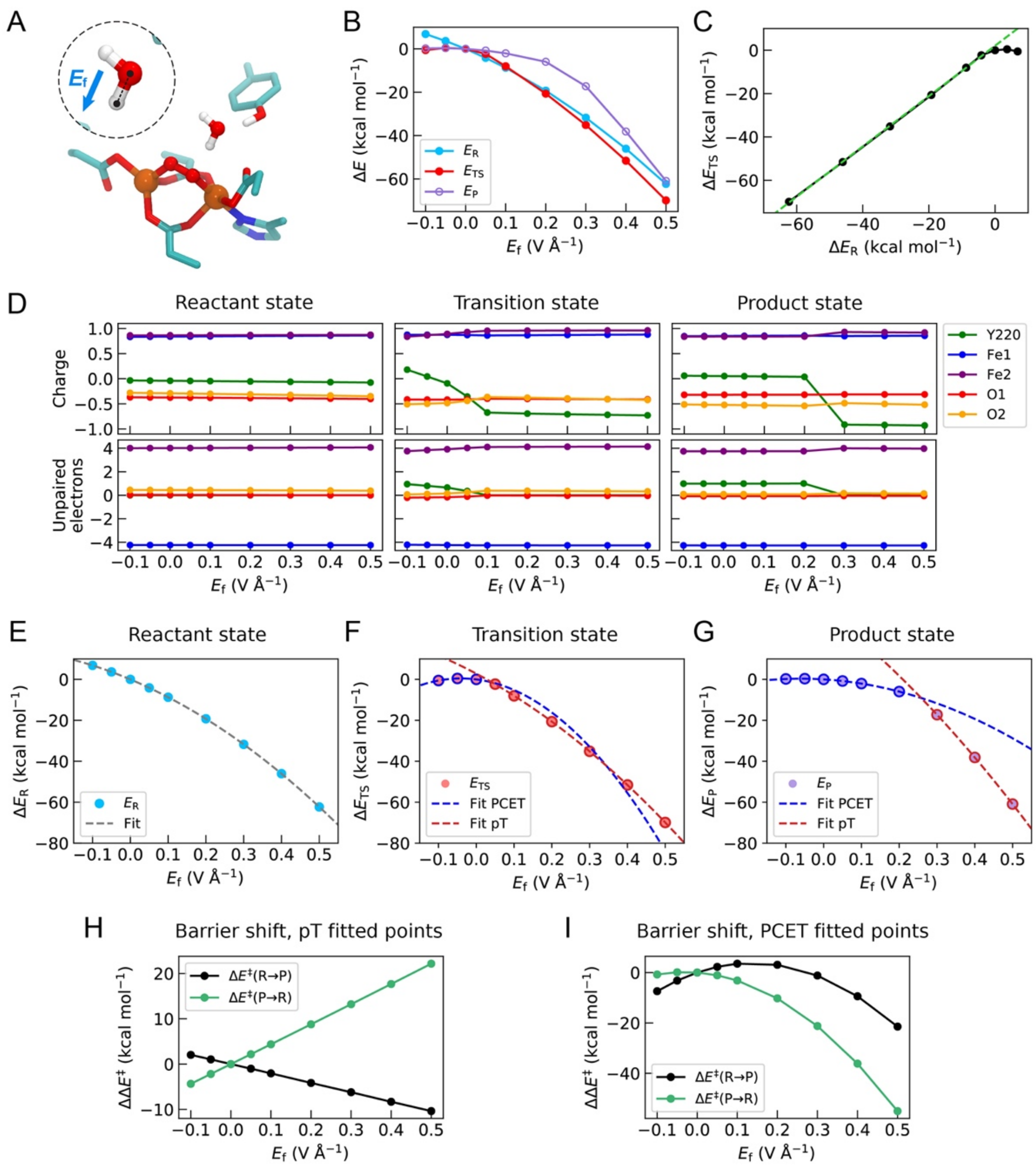

Figure S17. Electric field effects on the oxygen activation catalysis. A) A directed electric field (E_f, blue arrow) along the bridging water molecule between Tyr220 and the oxygen ligand, is applied to probe electric field effects on the PCET reaction. The E_f is applied on the optimized DFT models of state II (reactant, R), the transition state (TS), and state IIIb (product, P), optimized along the PCET pathway 2 (see main text). **B**) Relative shift of the R, TS, and P energies with the applied E_f. **C)** The relative shifts of R and TS energies with changing E_f show a linear slope of one (k~1.16). **D)** Effect of the E_f on the charge (*top panel*) and spin (*bottom panel*) distributions in R, TS, and P. For the R state, the external field does not change the electronic state. For TS, the E_f induces electron transfer from Tyr220 to the di-iron for $E_f < 0$, while for $E_f > 0$, the field results in a pure proton transfer reaction. For the P state, $E_f > 0$ induces a back-transfer of the electron from the di-iron to Tyr220, resulting in a tyrosinate species. **E-G)** Relative energy shifts of the R, TS, and P states with the applied field for the PCET and proton transfer regimes. **H-I**) Relative shift of the forward energy barrier (R→P, $\Delta E^{\ddagger}_{R\rightarrow P} = E_{TS} - E_R$) and the backward barrier ($\Delta E^{\ddagger}_{P\rightarrow R} = E_{TS} - E_P$) with the applied external E_f. Shift of $\Delta E^{\ddagger}$ vs. E_f for **H)** the proton transfer regime (linear fit) and **I)** the PCET regime (quadratic fit) of the reaction.

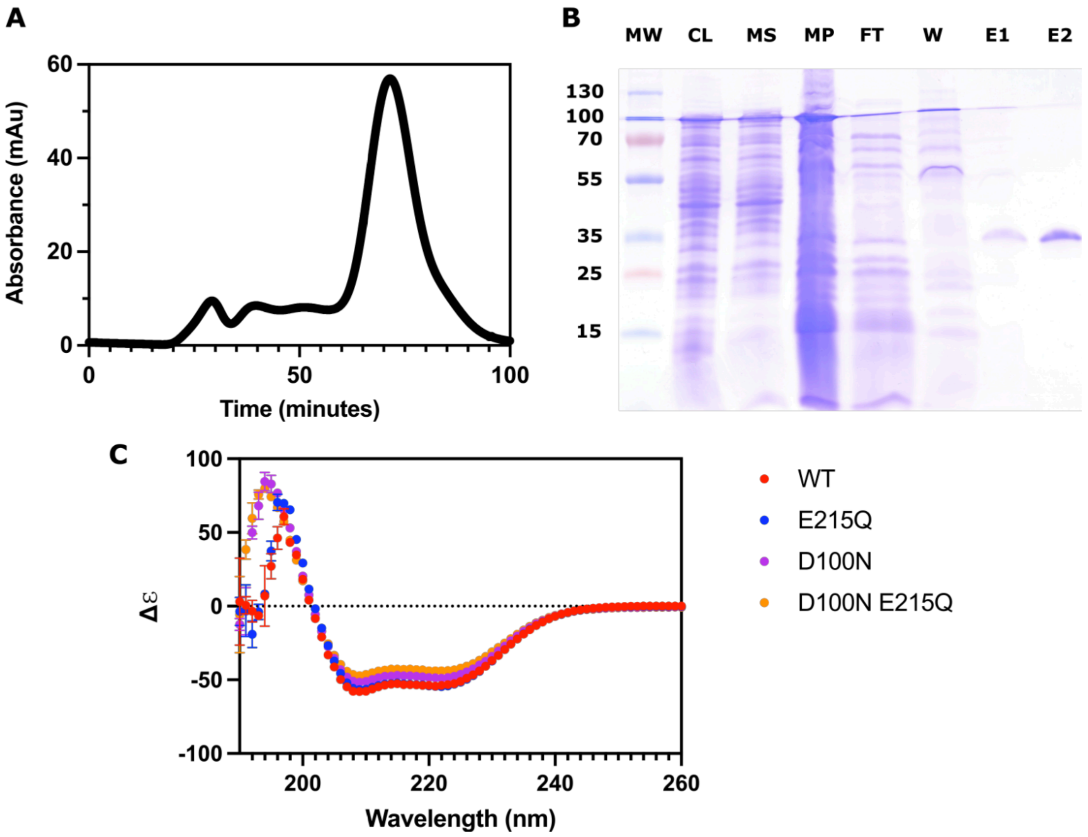

Figure S18. Purification and characterization of WT and mutant constructs of AOX. A) Size-exclusion chromatography profile of AOX performed on a HiLoad 16/600 Superdex 200 pg column. **B)** Purified AOX on SDS-PAGE gel, MW: molecular weight ladder, CL: cell lysate, MS: supernatant, MP: membranes, FT: flow through, W: wash, E1: elution fraction from His-Trap, E2: elution fraction from SEC. **C**) CD spectra measured at 25°C for the purified WT-AOX (red), E215Q (blue), and D100N (purple) mutants, and the D100N/E215Q double mutant (orange).

Table S1. List of DFT models and modeled oxidation and ligand states.

Model	Residues/molecules
QM1 (*N*=137)	E123, N161, E162, H165, E213, Y220, W247, I262, D265, E266, H269, O_2, Fe1, Fe2, H_2O
QM2 (*N*=203)	R96, T219, L122, Y220, E123, E266, H165, D265, N161, H269, E162, E213, L212, A216, E215, QH_2, Fe1(ferryl), Fe2 (ferric hydroxide), H_2O
QM3 (*N*=205)	R96, T219, L122, Y220, E123, E266, H165, D265, N161, H269, E162, E213, L212, A216, E215, QH_2, Fe1(ferric hydroxide), Fe2 (ferric hydroxide), H_2O
QM4 (*N*=148)	R96, R118, T219, L122, Y220, A216, Q215, D100, $QH^{\bullet}$, 7 H_2O

QM state	Model	Starting structure	Oxidation states	Description
Y1	QM1	Crystal structure (PDB ID: 3VV9)[1]	$Fe1^{2+}$, $Fe2^{2+}$, Y220-OH	Oxygen unbound, water coordinated between Fe1 and Fe2.
Y2	QM1	Y1	$Fe1^{3+}$, $Fe2^{2+}$, Y220-OH	Oxygen bound to Fe1 (κ^1-binding mode).
Y3	QM1	Y2	$Fe1^{2.5+}$, $Fe2^{2.5+}$, Y220-OH	Oxygen bound to Fe1 and Fe2 (μ_2-binding mode).
Y4	QM1	Y3	$Fe1^{2.5+}$, $Fe2^{3+}$, Y220-$O^{\bullet}$	Peroxy state (μ_2-bound oxygen protonated)
Y5	QM1	Y3	$Fe1^{3.5+}$, $Fe2^{2.5+}$, Y220-OH	Oxygen split state with one oxygen bound between both iron atoms.
Y6	QM1	Y4/Y5	$Fe1^{4+}$, $Fe2^{3+}$, Y220-$O^{\bullet}$	Fully oxidized di-iron center with ferryl and ferric hydroxide species.
Y7	QM2	S1	$Fe1^{4+}$, $Fe2^{3+}$, QH_2, Y220-$O^{\bullet}$, $E215^{-}$	Quinol bound-state with fully oxidized di-iron center.
Y8	QM2	Y7	$Fe1^{3+}$, $Fe2^{3+}$, $QH^{\bullet}$, Y220-$O^{\bullet}$, $E215^{-}$	Semi-quinone radical after PCET to the ferryl.
Y9	QM2	Y8	$Fe1^{3+}$, $Fe2^{3+}$, Q, Y220-O^{-}, E215-H	Quinone state after proton transfer to E215. The remaining electron is transferred to the tyrosyl radical resulting in tyrosinate.
Y10	QM3	Y9	$Fe1^{3+}$, $Fe2^{3+}$, QH_2, Y220-OH, $E215^{-}$	Second quinol bound-state, with diferric iron center. Y220 re-protonated by E215.
Y11	QM3	Y10	$Fe1^{2+}$, $Fe2^{3+}$, $Q^{\bullet/-}$, Y220-OH, E215-H	Anionic semi-quinone radical after PCET to the ferric Fe1 and proton transfer to E215.
Y12	QM3	Y11	$Fe1^{2+}$, $Fe2^{2+}$, Q, Y220-OH, $E215^{-}$	Quinone state after electron transfer to the ferric Fe2. Dissociation of water ligand from Fe1, reprotonation of the bridging OH^{-} to water, and deprotonated $E215^{-}$.
Y13	QM4	S13	$QH^{\bullet}$, Y220-$O^{\bullet}$, $D100^{-}$	Semi-quinone radical state for the E215Q mutant.
Y14	QM4	Y13	$Q^{\bullet/-}$, Y220-$O^{\bullet}$, D100H	Anionic semi-quinone after proton transfer to D100 for the E215Q mutant.

Table S2. List of atomistic MD simulations.

Simulation	AOX variant	Active site	Ligand	Length (ns)
S1	WT	$Fe^{IV}=O^{2-}/Fe^{III}-OH^{-}/Y220-O^{\bullet}/E215^{-}$	QH_2	500
S2	WT	$Fe^{IV}=O^{2-}/Fe^{III}-OH^{-}/Y220-O^{\bullet}/E215^{-}$	QH_2	250
S3	WT	$Fe^{IV}=O^{2-}/Fe^{III}-OH^{-}/Y220-O^{\bullet}/E215^{-}$	QH_2	250
S4	WT	$Fe^{III}-OH^{-}/Fe^{III}-OH^{-}/Y220-O^{\bullet}/E215^{-}$	$QH^{\bullet}$	500
S5	WT	$Fe^{III}-OH^{-}/Fe^{III}-OH^{-}/Y220-O^{\bullet}/E215^{-}$	$QH^{\bullet}$	250
S6	WT	$Fe^{III}-OH^{-}/Fe^{III}-OH^{-}/Y220-O^{\bullet}/E215^{-}$	$QH^{\bullet}$	250
S7	WT	$Fe^{III}-OH^{-}/Fe^{III}-OH^{-}/Y220-O^{-}/E215-H$	Q	500
S8	WT	$Fe^{III}-OH^{-}/Fe^{III}-OH^{-}/Y220-O^{-}/E215-H$	Q	250
S9	WT	$Fe^{III}-OH^{-}/Fe^{III}-OH^{-}/Y220-O^{-}/E215-H$	Q	250
S10	WT	$Fe^{III}-OH^{-}/Fe^{III}-OH^{-}/Y220-OH/E215^{-}$	QH_2	100
S11	WT	$Fe^{III}-OH^{-}/Fe^{III}-OH^{-}/Y220-OH/E215^{-}$	QH_2	100
S12	WT	$Fe^{IV}=O^{2-}/Fe^{III}-OH^{-}/Y220-O^{\bullet}/E215^{-}$	apo	500
S13	E215Q	$Fe^{IV}=O/Fe^{III}-OH^{-}/Y220-O^{\bullet}/E215^{-}$	QH_2	500
S14	E215Q	$Fe^{IV}=O/Fe^{III}-OH^{-}/Y220-O^{\bullet}/E215^{-}$	QH_2	250
**TOTAL**				**4.45 μs**

Table S3. Derived force field parameters for the active site of AOX. RESP point charge parameters of the di-iron center. The RESP charges for the two di-iron oxidation states (Fe^{III}/Fe^{III} and Fe^{IV}/Fe^{III}) were derived from DFT calculations. *N/A* - not applicable.

	**Fe^{III}-OH^-/Fe^{III}-OH^-**		**Fe^{IV}=O^{2-}/Fe^{III}-OH^-**	
**Atom name**	**Atom type**	**Q_i (Q_{tot}=0)**	**Atom type**	**Q_i (Q_{tot}=0)**
1FE1	FE1	1.0798	FE1	0.7488
1FE2	FE2	0.7472	FE2	0.6347
1O1	O1	-0.8096	O1	-0.4406
1O2	O2	-0.5869	O2	-0.428
1HF1	HO1	0.429	*N/A*	*N/A*
1HF2	HO2	0.3461	HO2	0.3603
2CB	CT2A	-0.2515	CT2A	-0.2462
2HB1	HA2	0.09	HA2	0.09
2HB2	HA2	0.09	HA2	0.09
2CG	CT2	0.0604	CT2	0.0321
2HG1	HA2	0.0231	HA2	0.0331
2HG2	HA2	0.0231	HA2	0.0331
2CD	CC2	0.7218	CC2	0.7054
2OE1	OC12	-0.5114	OC12	-0.4686
2OE2	OC22	-0.6032	OC22	-0.5634
3CB	CT2A	-0.2586	CT2A	-0.2495
3HB1	HA2	0.09	HA2	0.09
3HB2	HA2	0.09	HA2	0.09
3CG	CT2	-0.084	CT2	-0.0046
3HG1	HA2	0.0403	HA2	0.0174
3HG2	HA2	0.0403	HA2	0.0174
3CD	CC3	0.7394	CC3	0.6553
3OE1	OC13	-0.6816	OC13	-0.4951
3OE2	OC23	-0.5749	OC23	-0.5604
4CB	CT2A	-0.1328	CT2A	-0.1391
4HB1	HA2	0.09	HA2	0.09
4HB2	HA2	0.09	HA2	0.09
4CG	CT2	-0.2371	CT2	-0.2635
4HG1	HA2	0.0711	HA2	0.0958
4HG2	HA2	0.0711	HA2	0.0958
4CD	CC4	0.8608	CC4	0.7139
4OE1	OC14	-0.5949	OC14	-0.5364
4OE2	OC24	-0.5258	OC24	-0.3946
5CB	CT2A	-0.194	CT2A	-0.1842
5HB1	HA2	0.09	HA2	0.09
5HB2	HA2	0.09	HA2	0.09
5CG	CT2	-0.2362	CT2	-0.2393
5HG1	HA2	0.0817	HA2	0.0905
5HG2	HA2	0.0817	HA2	0.0905
5CD	CC5	1.0951	CC5	1.0279
5OE1	OC15	-0.6777	OC15	-0.6437
5OE2	OC25	-0.6761	OC25	-0.6482
6CB	CT2	-0.2064	CT2	-0.1975
6HB1	HA2	0.09	HA2	0.09
6HB2	HA2	0.09	HA2	0.09
6ND1	NR2	0.0242	NR2	0.0729
6CG	CPH1	0.1474	CPH1	0.1354
6CE1	CPH2	-0.0803	CPH2	-0.124
6HE1	HR1	0.1939	HR1	0.2103
6NE2	NR1	-0.1556	NR1	-0.151
6HE2	H	0.3634	H	0.3642
6CD2	CPH1	-0.264	CPH1	-0.2554
6HD2	HR3	0.2014	HR3	0.1985

Table S3 (contd.) Derived force field parameters for the active site of AOX. Lennard-Jones potentials of the di-iron center. The Lennard-Jones parameters were adapted from analogue atom types in the CHARMM36 force field.

**Atom type**	**ε_{ij} (kcal mol^{-1})**	**R_{min}/2 (Å)**
FE1	-0.25	0.65
FE2	-0.25	0.65
O1	-0.12	1.7
O2	-0.12	1.7
HO1	-0.046	0.2245
HO2	-0.046	0.2245
CC2	-0.07	2
CC3	-0.07	2
CC4	-0.07	2
CC5	-0.07	2
OC12	-0.12	1.7
OC22	-0.12	1.7
OC13	-0.12	1.7
OC23	-0.12	1.7
OC14	-0.12	1.7
OC24	-0.12	1.7
OC15	-0.12	1.7
OC25	-0.12	1.7

Table S3 (contd.) Derived force field parameters for the active site of AOX. Bonded parameters of the di-iron center: the di-iron center CHARMM36 bonded parameters were derived from DFT calculations. *N/A* - not applicable.

	Fe^{III}-OH^-/Fe^{III}-OH^-		Fe^{IV}=O^{2-}/Fe^{III}-OH^-	
**Bonds**	***k*** **(kcal mol^{-1} Å^{-2})**	r_0 **(Å)**	***k*** **(kcal mol^{-1} Å^{-2})**	r_0 **(Å)**
FE2-O2	84.4592	1.939	69.3257	1.962
FE1-O1	172.7962	1.833	387.2724	1.89
FE1-O2	80.4578	1.957	87.8505	1.945
HO2-O2	406.6297	1.005	387.2724	1.013
HO1-O1	450.3728	0.993	*N/A*	*N/A*
CT2-CC2	197.1315	1.516	201.6242	1.512
CC2-OC12	422.6099	1.283	432.852	1.28
CC2-OC22	531.6757	1.256	519.8372	1.259
OC12-FE2	33.1173	2.01	34.3714	2.01
OC22-FE1	28.6357	2.102	34.3714	2.069
CT2-CC3	188.3862	1.52	192.9973	1.519
CC3-OC13	440.2408	1.281	406.0952	1.29
CC3-OC23	595.5531	1.244	618.5185	1.238
OC13-FE1	63.7897	1.98	63.7898	1.951
CT2-CC4	200.3578	1.513	204.5685	1.509
CC4-OC14	484.6242	1.266	496.8762	1.263
CC4-OC24	490.7674	1.264	484.024	1.266
OC14-FE2	41.3254	1.999	43.8187	1.995
OC24-FE1	32.9086	2.044	41.6239	2.017
CT2-CC5	219.5622	1.499	223.7927	1.496
CC5-OC15	498.9304	1.269	495.9082	1.27
CC5-OC25	488.5964	1.27	482.1644	1.272
OC15-FE2	35.9	2.128	42.8318	2.072
OC25-FE2	30	2.09	40	2.1
NR2-FE2	51.653	2.117	50.1016	2.122

	Fe^{III}-OH^-/Fe^{III}-OH^-		Fe^{IV}=O^{2-}/Fe^{III}-OH^-	
**Angles**	***k*** **(kcal mol^{-1} rad^{-2})**	θ_0 **(deg)**	***k*** **(kcal mol^{-1} rad^{-2})**	θ_0 **(deg)**
FE2-O2-FE1	61.3166	123.576	50.6086	120.304
FE2-O2-HO2	50	115.612	31.7006	115.004
FE1-O1-HO1	50	122.413	N/A	N/A
FE1-O2-HO2	50	109.503	27.6702	115.237
O1-FE1-O2	44.0263	93.163	50	85.34
CT2A-CT2-CC2	140.1607	113.139	140.108	112.807
CT2-CC2-OC12	93.4937	116.871	93.5793	117.379
CT2-CC2-OC22	95.2847	120.14	94.7349	120.135
CC2-CT2-HA2	67.0919	107.854	67.0919	107.854
CC2-OC22-FE1	35.6596	124.025	29.0569	121.599
OC22-FE1-OC13	35.6765	94.596	35.6765	94.596
OC22-FE1-OC24	62.8785	117.884	50.405	112.977
OC22-FE1-O2	66.4823	81.95	50.7189	87.245
OC12-CC2-OC22	98.7874	122.982	98.0336	122.477
OC22-FE1-O1	58.7721	132.675	25	129.88
CC2-OC12-FE2	23.7085	124.079	30	126.72
OC12-FE2-O2	66.9961	86.03	30	86.84
OC12-FE2-OC14	52.9049	96.568	30	97.66
OC25-FE2-OC12	93.6152	103.447	30	102.95
OC15-FE2-OC12	30	164.59	30	165.23
OC12-FE2-NR2	44.6789	94.15	30	91.69
CT2A-CT2-CC3	113.6427	114.059	140.713	113.227
CT2-CC3-OC13	93.9686	116.039	85.3557	116.466
CT2-CC3-OC23	103.1638	119.466	92.5086	119.619
OC13-CC3-OC23	96.9446	124.494	90.5654	123.89
CC3-CT2-HA2	67.0919	107.854	71.023	108.58
CC3-OC13-FE1	30	137.82	43.4211	134.865
OC13-FE1-O1	30	97.89	25	86.99
OC13-FE1-O2	30	168.1	25.6881	171.196
OC13-FE1-OC24	30	90.747	37.1515	95.407
CT2A-CT2-CC4	134.0015	113.808	108.181	113.38
CT2-CC4-OC14	89.175	115.47	93.1509	115.799
CT2-CC4-OC24	92.2871	118.94	97.7913	118.753

CC4-CT2-HA2	67.0919	107.854	68.4288	106.696
OC14-CC4-OC24	98.9602	125.589	97.8774	125.436
OC24-FE1-O1	55.8603	109.09	25	116.73
OC24-FE1-O2	62.2082	89.795	46.8775	91.78
CC4-OC14-FE2	51.6171	132.808	44.6848	130.686
CC4-OC24-FE1	28.2934	135.586	49.7331	135.56
OC14-FE2-O2	48.9766	92.843	49.217	91.908
OC15-FE2-OC14	81.6199	97.59	81.6199	96.519
NR2-FE2-OC14	45.6034	87.721	42.4977	86.604
CT2A-CT2-CC5	119.035	111.527	122.24	111.379
CT2-CC5-OC15	107.1604	120.91	109.569	121.183
CT2-CC5-OC25	111.0791	120.79	113.666	120.958
CC5-CT2-HA2	67.0919	107.854	69.5514	107.979
CC5-OC15-FE2	32.0014	90.12	32.0014	90.043
OC15-FE2-NR2	40.7201	92.47	40.7201	93.248
OC15-FE2-O2	51.0669	87.21	51.0669	88.582
OC15-CC5-OC25	114.5989	118.272	116.801	117.834
CC5-OC25-FE2	42.679	88.392	30	88.57
OC25-FE2-O2	41.9335	85.758	50	87.64
OC25-FE2-OC14	57.714	159.776	40	159.33
OC25-FE2-NR2	36.5585	93.628	40	94.34
CPH1-NR2-FE2	35.27	133.576	35.1344	133.674
CPH2-NR2-FE2	32.887	118.909	33.7	118.787
NR2-FE2-O2	44.9816	179.385	47.6531	177.76

	Fe^{III}-OH^{-}/Fe^{III}-OH^{-}		Fe^{IV}=O^{2-}/Fe^{III}-OH^{-}	
**Impropers**	***k* (kcal mol^{-1} rad^{-2})**	**χ_0 (deg)**	***k* (kcal mol^{-1} rad^{-2})**	**χ_0 (deg)**
CC5-CT2-OC25-OC15	1.32	1.051	1.32	1.051
CC2-CT2-OC22-OC12	1.25	-0.59	1.25	-0.59
CC3-CT2-OC23-OC13	0.47	-0.953	0.47	-0.953
CC4-CT2-OC24-OC14	0.38	0.679	0.0197	0.679
CC5-CT2-OC15-OC25	0.0467	1.106	0.0419	1.051
CC2-CT2-OC12-OC22	0.0135	0.516	0.0175	0.59
NR2-CPH1-CPH2-FE2	1.9078	6.56	2.1833	6.955
CC3-CT2-OC13-OC23	0.0009	0.141	0.0416	0.953
CC4-CT2-OC14-OC24	0.0008	0.139	0.38	0.679
O2-FE2-FE1-HO2	0.53	18.669	12.2531	16.06

	Fe^{III}-OH^{-}/Fe^{III}-OH^{-}			Fe^{IV}=O^{2-}/Fe^{III}-OH^{-}		
**Dihedrals**	***k* (kcal mol^{-1})**	**n**	**δ**	***k* (kcal mol^{-1})**	**n**	**δ**
FE2-O2-FE1-O1	3	1	23.08	1.5	1	17.74
O2-FE1-O1-HO1	3	1	103.24	*N/A*	*N/A*	*N/A*
O1-FE1-O2-HO2	3	1	164.85	1.5	1	162
OC24-FE1-O1-HO1	1	1	12.36	*N/A*	*N/A*	*N/A*
OC24-FE1-O2-HO2	1	1	-86.04	*N/A*	*N/A*	*N/A*
OC22-FE1-O1-HO1	1	1	-174.8	*N/A*	*N/A*	*N/A*
OC22-FE1-O2-HO2	1	1	32.18	*N/A*	*N/A*	*N/A*
OC14-FE2-O2-HO2	1	1	89.68	*N/A*	*N/A*	*N/A*
CT1-CT2A-CT2-CC2	0.19	3	0	0.19	3	0
HA2-CT2A-CT2-CC2	0.19	3	0	0.19	3	0
HA2-CT2-CC2-OC22	0.2	3	78.59	0.2	3	78
HA2-CT2-CC2-OC12	0.2	3	-78.86	0.2	3	-81
CT2A-CT2-CC2-OC12	0.6	1	-151.14	0.6	1	180
CT2A-CT2-CC2-OC22	0.6	1	27.92	0.6	1	0
CT2-CC2-OC22-FE1	0.2	1	-21.47	0.3	1	-20
CC2-OC22-FE1-O1	0.2	1	56.32	0.1	1	61
CC2-OC22-FE1-O2	0.4	1	143.19	0.4	1	143
CC2-OC22-FE1-OC13	0.2	1	-41.93	0.2	1	-29
CC2-OC22-FE1-OC24	0.3	1	-131.34	0.3	1	-127
CT2-CC2-OC12-FE2	0.3	1	67.89	0.2	1	67.84
CC2-OC12-FE2-O2	0.3	1	150.88	0.4	1	149.61
CC2-OC12-FE2-OC14	0.4	1	58.46	0.2	1	58.11
OC25-FE2-OC12-CC2	0.2	1	-124.45	0.2	1	-123.54
CC2-OC12-FE2-OC15	0.2	1	-144.93	0.2	1	-138.24
CC2-OC12-FE2-NR2	0.2	1	-29.72	0.2	1	-28.69
CT1-CT2A-CT2-CC3	0.19	3	0	0.19	3	0

HA2-CT2A-CT2-CC3	0.19	3	0	0.19	3	0
CT2A-CT2-CC3-OC13	0.6	1	4.35	0.6	1	-24
CT2A-CT2-CC3-OC23	0.6	1	-175.91	0.6	1	155
HA2-CT2-CC3-OC13	0.2	3	2.38	0.2	3	-87
HA2-CT2-CC3-OC23	0.2	3	-154.49	0.2	3	-252
CT2-CC3-OC13-FE1	0.2	1	56.21	0.2	1	29
CC3-OC13-FE1-O1	0.2	1	-67.61	0.1	1	-39
CC3-OC13-FE1-O2	0.2	1	90.47	0.1	1	-11
CC3-OC13-FE1-OC24	0.2	1	-177	0.3	1	-156
OC22-FE1-OC13-CC3	0.2	1	65.12	0.3	1	90
CT1-CT2A-CT2-CC4	0.19	3	0	0.19	3	0
HA2-CT2A-CT2-CC4	0.19	3	0	0.19	3	0
CT2A-CT2-CC4-OC14	0.6	1	-28.4	0.6	1	0
CT2A-CT2-CC4-OC24	0.6	1	151.86	0.6	1	180
HA2-CT2-CC4-OC14	0.2	3	-99.49	0.2	3	0
HA2-CT2-CC4-OC24	0.2	3	-256.63	0.2	3	180
CT2-CC4-OC14-FE2	0.2	1	1.48	0.2	1	9
CT2-CC4-OC24-FE1	0.2	1	0	0.2	1	8
CC4-OC14-FE2-O2	0.3	1	157.12	0.4	1	144
CC4-OC24-FE1-O1	0.2	1	-65.298	0.1	1	-85
CC4-OC24-FE1-O2	0.3	1	-158.58	0.3	1	-170
OC13-FE1-OC24-CC4	0.2	1	33.32	0.1	1	4
OC22-FE1-OC24-CC4	0.3	1	120.65	0.3	1	102
OC15-FE2-OC14-CC4	0.2	1	69.55	0.3	1	55
NR2-FE2-OC14-CC4	0.2	1	-22.641	0.3	1	-38
OC25-FE2-OC14-CC4	*N/A*	*N/A*	*N/A*	0.3	1	55.27
CT1-CT2A-CT2-CC5	0.19	3	0	0.19	3	0
HA2-CT2A-CT2-CC5	0.19	3	0	0.19	3	0
CT2A-CT2-CC5-OC15	0.5	1	-3.36	0.5	1	-3
CT2A-CT2-CC5-OC25	0.5	1	178.64	0.5	1	179
HA2-CT2-CC5-OC15	0.3	3	-18.65	0.2	3	0
HA2-CT2-CC5-OC25	0.3	3	-172.61	0.2	3	180
CT2-CC5-OC15-FE2	0.2	1	11.84	0.4	1	10
CC5-OC15-FE2-NR2	0.2	1	81.33	0.3	1	82
CC5-OC15-FE2-OC14	0.2	1	-6.7	0.4	1	-5
CC5-OC15-FE2-O2	0.2	1	-99.2	0.3	1	-97
CT2-CC5-OC25-FE2	0.3	1	-11.66	0.4	1	-10.13
CC5-OC25-FE2-O2	0.4	1	96.79	0.3	1	95.44
CC5-OC25-FE2-OC14	0.1	1	-176.5	0.4	1	-175.41
CC5-OC25-FE2-NR2	0.2	1	-83.24	0.4	1	-83.52
CC5-OC25-FE2-OC12	0.4	1	11.91	0.4	1	9.23
CC5-OC15-FE2-OC12	N/A	N/A	N/A	0.3	1	-168.92
CPH2-NR2-FE2-OC14	0.3	1	141.21	0.4	1	140
CPH2-NR2-FE2-O2	0.1	1	-15.41	0.1	1	-172
OC15-FE2-NR2-CPH2	0.2	1	-149.99	0.8	1	43
FE2-NR2-CPH2-NR1	0.2	1	-10.41	0.2	1	-11

Table S4. Primers for the E215Q and D100N variants.

	Primer sequence (5’ → 3’)
AOX E215Q Forward	GGTTACCTTGAAGAGCAAGCCGTCATTACATACACCGG
AOX E215Q Reverse	CTCTTCAAGGTAACCGACAAAGC
AOX D100N Forward	CCTGCCGCTGGTTATTTAACACATTCTCTCTCTACCGTTTCGGTTCCA
AOX D100N Reverse	AAATAACCAGCGGCAGGTGCGG

Table S5. Benchmarking the DFT calculations for a di-radical model. The table shows electronic energies at the DFT and RPA[13] levels for a model system comprising protein residues, water molecules, and the $QH^{\bullet}$ as well as Tyr-$O^{\bullet}$ radical species, but excluding the di-iron core (*N*=148 atoms, see Figure S15). The calculations were performed at the def2-TZVPP/ε=4 level of theory. See also Ref. 17, suggesting that ωB97X-D and CASSCF/NEVPT2 predict similar barriers for PCET along Tyr radicals in ribonucleotide reductase.

System (*N*=148)	B3LYP	ωB97X-D	RPA
I	0.0	0.0	0.0
TS I-II	15.4	17.8	18.4
II	9.9	10.7	11.0

Table S6. Benchmarking the DFT calculations for the quinol oxidation reaction. The table shows electronic energies at the DFT and RPA[13] levels for a model system comprising the QH_2, surrounding protein residues, and the iron core with *N*=160 atoms. The model was constructed based on the larger DFT model with *N*=203, benchmarked using different density functionals. The calculations were performed at the def2-TZVP/ε=4 (*N*=203 atoms) and def2-TZVPP/ε=4 (*N*=160 atoms) levels. See Figure S6 and S15 for the model systems.

System (*N*=203)	B3LYP/ def2-TZVP	B3LYP*/ def2-TZVP	CAM-B3LYP/ def2-TZVP	CAMh-B3LYP/ def2-TZVP	ωB97X-D /def2-TZVP
I	0.0	0.0	0.0	0.0	0.0
TS	2.6	2.7	8.7	6.8	8.8
II	-29.7	-24.1	-34.8	-33.2	-34.2

System (*N*=160)	B3LYP/ def2-TZVPP	RPA/ def2-TZVPP
I	0.0	0.0
TS	2.5	6.0
II	-29.5	-25.9

Table S7. Benchmarking the DFT calculations for the oxygen splitting reaction. The table shows electronic energies at the DFT levels for a model system of the oxygen splitting reaction comprising the di-iron core and Tyr220 (*N*=137 atoms). The calculations were performed at the def2-TZVP/ε=4 levels. See Figure S15 for the model system.

**System (*N*=137)/ PCET + O-O split**	**B3LYP/ def2-TZVP**	**B3LYP*/ def2-TZVP**	**CAM-B3LYP/ def2-TZVP**	**CAMh-B3LYP/ def2-TZVP**	**ωB97X-D/ def2-TZVP**
I	0.0	0.0	0.0	0.0	0.0
TS I-II	8.2	5.3	9.7	8.6	9.5
II	-10.7	-13.3	-8.6	-9.8	-7.8
TS II-IIIa	10.5	5.4	11.5	11.6	16.0
IIIa	-0.6	-0.4	-2.6	0.6	0.8
TS IIIa-IV	8.2	8.1	7.0	9.7	11.3
IV	-28.8	-35.0	-22.2	-26.0	-18.1

**System (*N*=137)/ O-O split + PCET**	**B3LYP/ def2-TZVP**	**B3LYP*/ def2-TZVP**	**CAM-B3LYP/ def2-TZVP**	**CAMh-B3LYP/ def2-TZVP**	**ωB97X-D/ def2-TZVP**
I	0.0	0.0	0.0	0.0	0.0
TS I-II	8.2	5.3	9.7	8.6	9.5
II	-10.7	-13.3	-8.6	-9.8	-7.8
TS II-IIIb	5.3	-1.8	15.0	6.6	16.8
IIIb	-3.9	-10.9	5.4	-1.8	8.3
TS IIIb-IV	-19.2	-26.3	-12.4	-16.6	-7.3
IV	-28.8	-35.0	-22.2	-26.0	-18.1

SI References

1 T. Shiba, Y. Kido, K. Sakamoto, D. K. Inaoka, C. Tsuge, R. Tatsumi, G. Takahashi, E. O. Balogun, T. Nara, T. Aoki, T. Honma, A. Tanaka, M. Inoue, S. Matsuoka, H. Saimoto, A. L. Moore, S. Harada and K. Kita, *Proc. Natl. Acad. Sci.*, 2013, **110**, 4580–4585.
2 E. Chovancova, A. Pavelka, P. Benes, O. Strnad, J. Brezovsky, B. Kozlikova, A. Gora, V. Sustr, M. Klvana, P. Medek, L. Biedermannova, J. Sochor and J. Damborsky, *PLoS Comput. Biol.*, 2012, **8**, e1002708.
3 J. C. Phillips, D. J. Hardy, J. D. C. Maia, J. E. Stone, J. V. Ribeiro, R. C. Bernardi, R. Buch, G. Fiorin, J. Hénin, W. Jiang, R. McGreevy, M. C. R. Melo, B. K. Radak, R. D. Skeel, A. Singharoy, Y. Wang, B. Roux, A. Aksimentiev, Z. Luthey-Schulten, L. V. Kalé, K. Schulten, C. Chipot and E. Tajkhorshid, *J. Chem. Phys.*, 2020, **153**, 044130.
4 R. Best, X. Zhu, J. Shim, P. Lopes, J. Mittal, M. Feig and A. MacKerell, *J. Chem. Theory Comput.*, 2012, **8**, 3257–3273.
5 A. D. Becke, *J. Chem. Phys.*, 1993, **98**, 5648–5652.
6 C. Lee, W. Yang and R. G. Parr, *Phys. Rev. B*, 1988, **37**, 785–789.
7 S. Grimme, J. Antony, S. Ehrlich and H. Krieg, *J. Chem. Phys.*, 2010, **132**, 154104.
8 A. Schäfer, H. Horn and R. Ahlrichs, *J. Chem. Phys.*, 1992, **97**, 2571–2577.
9 A. Klamt and G. Schüürmann, *J Chem Soc Perkin Trans 2*, 1993, 799–805.
10 A. Klamt, V. Jonas, T. Bürger and J. C. W. Lohrenz, *J. Phys. Chem. A*, 1998, **102**, 5074–5085.
11 Louis. Noodleman, D. A. Case and Arie. Aizman, *J. Am. Chem. Soc.*, 1988, **110**, 1001–1005.
12 J.-M. Mouesca, L. Noodleman and D. A. Case, *Int. J. Quantum Chem.*, 1995, **56**, 95–102.
13 F. Furche, *Phys. Rev. B*, 2001, **64**, 195120.
14 S. G. Balasubramani, G. P. Chen, S. Coriani, M. Diedenhofen, M. S. Frank, Y. J. Franzke, F. Furche, R. Grotjahn, M. E. Harding, C. Hättig, A. Hellweg, B. Helmich-Paris, C. Holzer, U. Huniar, M. Kaupp, A. Marefat Khah, S. Karbalaei Khani, T. Müller, F. Mack, B. D. Nguyen, S. M. Parker, E. Perlt, D. Rappoport, K. Reiter, S. Roy, M. Rückert, G. Schmitz, M. Sierka, E. Tapavicza, D. P. Tew, C. van Wüllen, V. K. Voora, F. Weigend, A. Wodyński and J. M. Yu, *J. Chem. Phys.*, 2020, **152**, 184107.
15 Grossfield, A, http://membrane.urmc.rochester.edu/wordpress/?page_id=126.
16 B. Brooks, C. Brooks, A. Mackerell, L. Nilsson, R. Petrella, B. Roux, Y. Won, G. Archontis, C. Bartels, S. Boresch, A. Caflisch, L. Caves, Q. Cui, A. Dinner, M. Feig, S. Fischer, J. Gao, M. Hodoscek, W. Im, K. Kuczera, T. Lazaridis, J. Ma, V. Ovchinnikov, E. Paci, R. Pastor, C. Post, J. Pu, M. Schaefer, B. Tidor, R. Venable, H. Woodcock, X. Wu, W. Yang, D. York and M. Karplus, *J. Comput. Chem.*, 2009, **30**, 1545–1614.
17 S. Riahi and C. N. Rowley, *J. Comput. Chem.*, 2014, **35**, 2076–2086.
18 L. Young, A. Rosell-Hidalgo, D. K. Inaoka, F. Xu, M. Albury, B. May, K. Kita and A. L. Moore, *Biochim. Biophys. Acta BBA - Bioenerg.*, 2020, **1861**, 148247.
19 Y. Kido, K. Sakamoto, K. Nakamura, M. Harada, T. Suzuki, Y. Yabu, H. Saimoto, F. Yamakura, D. Ohmori, A. Moore, S. Harada and K. Kita, *Biochim. Biophys. Acta BBA - Bioenerg.*, 2010, **1797**, 443–450.
20 C. Affourtit and A. L. Moore, *Biochim. Biophys. Acta BBA - Bioenerg.*, 2004, **1608**, 181–189.
21 M. Reiher, O. Salomon and B. Artur Hess, *Theor. Chem. Acc. Theory Comput. Model. Theor. Chim. Acta*, 2001, **107**, 48–55.
22 T. Yanai, D. P. Tew and N. C. Handy, *Chem. Phys. Lett.*, 2004, **393**, 51–57.
23 Y. Shao, Y. Mei, D. Sundholm and V. R. I. Kaila, *J Chem Theory Comput*, 2020, **16**, 587–600.
24 J. Tao, J. P. Perdew, V. N. Staroverov and G. E. Scuseria, *Phys. Rev. Lett.*, 2003, **91**, 146401.
25 J.-D. Chai and M. Head-Gordon, *Phys. Chem. Chem. Phys.*, 2008, **10**, 6615.
26 T. Shiba, D. K. Inaoka, G. Takahashi, C. Tsuge, Y. Kido, L. Young, S. Ueda, E. O. Balogun, T. Nara, T. Honma, A. Tanaka, M. Inoue, H. Saimoto, S. Harada, A. L. Moore and K. Kita, *Biochim. Biophys. Acta BBA - Bioenerg.*, 2019, **1860**, 375–382.
27 F. Sievers, A. Wilm, D. Dineen, T. J. Gibson, K. Karplus, W. Li, R. Lopez, H. McWilliam, M. Remmert, J. Söding, J. D. Thompson and D. G. Higgins, *Mol. Syst. Biol.*, 2011, **7**, 539.
